# High-Resolution Analyses of Human Leukocyte Antigens Allele and Haplotype Frequencies Based on 169,995 Volunteers from the China Bone Marrow Donor Registry Program

**DOI:** 10.1371/journal.pone.0139485

**Published:** 2015-09-30

**Authors:** Xiao-Yang Zhou, Fa-Ming Zhu, Jian-Ping Li, Wei Mao, De-Mei Zhang, Meng-Li Liu, Ai-Lian Hei, Da-Peng Dai, Ping Jiang, Xiao-Yan Shan, Bo-Wei Zhang, Chuan-Fu Zhu, Jie Shen, Zhi-Hui Deng, Zheng-Lei Wang, Wei-Jian Yu, Qiang Chen, Yan-Hui Qiao, Xiang-Ming Zhu, Rong Lv, Guo-Ying Li, Guo-Liang Li, Heng-Cong Li, Xu Zhang, Bin Pei, Li-Xin Jiao, Gang Shen, Ying Liu, Zhi-Hui Feng, Yu-Ping Su, Zhao-Xia Xu, Wen-Ying Di, Yao-Qin Jiang, Hong-Lei Fu, Xiang-Jun Liu, Xiang Liu, Mei-Zhen Zhou, Dan Du, Qi Liu, Ying Han, Zhi-Xin Zhang, Jian-Ping Cai

**Affiliations:** 1 The Key Laboratory of Geriatrics, Beijing hospital & Beijing Institute of Geriatrics, Ministry of Health, Beijing, China; 2 Quality control laboratory, China Bone Marrow Program, Beijing, China; 3 HLA Laboratory, Zhejiang Blood Center, Hangzhou, Zhejiang, China; 4 HLA Laboratory, Liaoning Blood Center, Shenyang, Liaoning, China; 5 HLA Laboratory, Chongqing Blood Center, Chongqing, China; 6 HLA Laboratory, Taiyuan Red Cross Blood Center, Taiyuan, Shanxi, China; 7 HLA Laboratory, Shaanxi Blood Center, Xi’an, Shaanxi, China; 8 HLA Laboratory, Beijing Red Cross Blood Center, Beijing, China; 9 HLA Laboratory, Henan Blood Center, Zhengzhou, Henan, China; 10 HLA Laboratory, Shandong Blood Center, Jinan, Shandong; 11 HLA Laboratory, the First Affiliated Hospital of Nanjing Medical University, Nanjing, Jiangsu, China; 12 The Key Laboratory of Histocompatibility and Immunogenetics, Shenzhen Blood Center, Shenzhen, Guangdong, China; 13 HLA Laboratory, Hebei Blood Center, Shijiazhuang, Hebei, China; 14 HLA Laboratory, Dalian Red Cross Blood Center, Dalian, Liaoning, China; 15 HLA Laboratory, Institute of Blood Transfusion, Chinese Academy of Medical Sciences & Peking Union Medical College, Chengdu, Sichuan, China; 16 HLA Laboratory, Xinjiang Blood Center, Urumchi, Xinjiang, China; 17 HLA Laboratory, Kunming Blood Center, Kunming, Yunnan, China; 18 HLA Laboratory, Hefei Red Cross Blood Center, Hefei, Anhui, China; 19 HLA Laboratory, Gansu Red Cross Blood Center, Lanzhou, Gansu, China; 20 HLA Laboratory, Jiangxi Blood Center, Nanchang, Jiangxi, China; 21 HLA Laboratory, Nanning Blood Center, Nanning, Guangxi, China; 22 HLA Laboratory, Xiamen Blood Center, Xiamen, Fujian, China; 23 HLA Laboratory, Changchun Blood Center, Changchun, Jilin, China; 24 HLA Laboratory, Wuhan Blood Center, Wuhan, Hubei, China; 25 HLA Laboratory, Harbin Red Cross Blood Center, Harbin, Heilongjiang, China; 26 HLA Laboratory, Qingdao Blood Center, Qingdao, Shandong, China; 27 HLA Laboratory, Yueyang Red Cross Blood Center, Yueyang, Hunan, China; 28 HLA Laboratory, Changsha Blood Center, Changsha, Hunan, China; 29 HLA Laboratory, Soochow Red Cross Blood Center, Suzhou, Jiangsu, China; 30 HLA Laboratory, Shanghai Blood Center, Shanghai, China; 31 HLA Laboratory, BFR Transplant Diagnostic Service Center, Beijing China; 32 HLA Laboratory, CapitalBio Corporation, Beijing, China; 33 HLA Laboratory, Beijing Genomics Institute, Shenzhen, Guangdong, China; 34 Department of HLA Technology, China Bone Marrow Program, Beijing, China; Centro di Riferimento Oncologico, IRCCS National Cancer Institute, ITALY

## Abstract

Allogeneic hematopoietic stem cell transplantation is a widely used and effective therapy for hematopoietic malignant diseases and numerous other disorders. High-resolution human leukocyte antigen (HLA) haplotype frequency distributions not only facilitate individual donor searches but also determine the probability with which a particular patient can find HLA-matched donors in a registry. The frequencies of the *HLA-A*, *-B*, *-C*, *-DRB1*, *and -DQB1* alleles and haplotypes were estimated among 169,995 Chinese volunteers using the sequencing-based typing (SBT) method. Totals of 191 *HLA-A*, 244 *HLA-B*, 146 *HLA*-C, 143 *HLA-DRB1* and 47 *HLA-DQB1* alleles were observed, which accounted for 6.98%, 7.06%, 6.46%, 9.11% and 7.91%, respectively, of the alleles in each locus in the world (IMGT 3.16 Release, Apr. 2014). Among the 100 most common haplotypes from the 169,995 individuals, nine distinct haplotypes displayed significant regionally specific distributions. Among these, three were predominant in the South China region (i.e., the 20^th^, 31^st^, and 81^st^haplotypes), another three were predominant in the Southwest China region (i.e., the 68^th^, 79^th^, and 95^th^ haplotypes), one was predominant in the South and Southwest China regions (the 18^th^ haplotype), one was relatively common in the Northeast and North China regions (the 94^th^ haplotype), and one was common in the Northeast, North and Northwest China (the 40^th^ haplotype). In conclusion, this is the first to analyze high-resolution HLA diversities across the entire country of China, based on a detailed and complete data set that covered 31 provinces, autonomous regions, and municipalities. Specifically, we also evaluated the HLA matching probabilities within and between geographic regions and analyzed the regional differences in the HLA diversities in China. We believe that the data presented in this study might be useful for unrelated HLA-matched donor searches, donor registry planning, population genetic studies, and anthropogenesis studies.

## Introduction

The human leukocyte antigen (HLA) system is well known as a highly polymorphic genetic system. The main function of HLA molecules is to present antigenic peptides to the immune system and thus regulate the induction of immune responses [1,2]. As a consequence of many features, HLA antigens have an important influence on the outcome of hemopoietic stem cell transplantation (HSCT) [3]. Allogeneic hematopoietic stem cell transplantation (HSCT) is a well-established therapy for hematologic and lymphoid cancers and numerous other disorders [4–6]. In recent years, patients undergoing allogeneic HSCT have greatly benefited from the deeper understanding of the HLA system and particularly from high-resolution definition of HLA class I and HLA class II transplantation antigens [7]. Donor-recipient HLA matching at the allelic level is required for the success of unrelated donor marrow transplantation. Furthermore, population-specific high-resolution HLA haplotype frequency distributions not only facilitate individual donor searches but also determine the chance with which a particular patient can find fully matched donors in a registry [8–10].

The Chinese Bone Marrow Donor Program (CMDP) manages countrywide unrelated hematopoietic stem cell donor recruitment, maintenance and clinical utilities. It has 31 active provincial branch registry networks. By the end of 2013, the CMDP had enlisted 1.83 million potential HSCT donors and facilitated more than 3,900 HSCT donations including 133 for patients beyond Mainland China. Due to the high HLA diversity in the Chinese population, many patients in need of a hematopoietic stem cell transplant are still currently unable to find fully matched donors at the five loci of *HLA-A*, *-B*, *-C*, *-DRB1*, and-*DQB1*. Consequently, there are clear needs to increase the diversity of the donor pool and improve the unrelated donor search strategies. Beginning in 2009, the CMDP performed the HLA typing of a certain percentage of newly recruited volunteers at the high-resolution level (two fields). As HLA typing techniques have evolved [11,12], the CMDP has accumulated a large data set of high-resolution *HLA-A*, *-B*, *-C*, *-DRB1* and -*DQB1* typing. Over the last ten years, numerous papers have been published about HLA allele and haplotype distributions in the Chinese population. However, in those studies, the recruited potential donors were most frequently from only one or several provinces [13–24] or from several ethnic populations [25–34]. Until now, no one has explored the regional differences and characteristics of HLA diversity at the allele level across the entire country.

There are 1.37 billion people living in China who account for one fifth of the total population of the world [35]. The population distribution is extremely uneven across the vast territory of China [36]. Moreover, the Chinese population consists of 56 ethnic groups, the largest of which is the Han Chinese, which constitutes approximately 91.51% of the total population [35], and the ethnic minorities account for approximately 8.49% of the total population according to the Sixth National Population Census of the People’s Republic of China (2010, November) [35]. Therefore, China has an abundant, complicated and valuable resource of human genetic diversity.

Our study is the first attempt to analyze high-resolution HLA diversity in China at the countrywide scale; we covered 31 provinces, autonomous regions, and municipalities. Our primary goal in this initial study was to determine the regional differences and characteristics of HLA diversities across seven geographical regions of China. Our results might be useful for strategic planning of donor recruitment in China, for population genetics research, and for anthropological and disease association studies.

## Results

### HLA allele frequencies

The numbers of observed alleles for the different *HLA* genes were 191 for *HLA-A*, 244 for *HLA-B*, 146 for *HLA-C*, 143 for *HLA-DRB1* and 47 for *HLA-DQB1*, which accounted for 6.98% for *HLA-A*, 7.06% for *HLA-B*, 6.46% for *HLA-C*, 9.11% for *HLA-DRB1* and 7.91% for *HLA-DQB1*, respectively, in the IMGT/HLA Database release 3.16 (http://www.ebi.ac.uk/ipd/imgt/hla/stats.html) (Table 1). The allele frequencies are displayed in S1–S5 Tables. The greatest allelic diversity occurs for *HLA-B*, and the least diversity is present in *HLA-DQB1*.

**Table 1:** Numbers of HLA alleles observed in 169,995 Chinese individuals.

	Number of alleles in China	Number of alleles in the World ^a^ (IMGT)	Percent
**HLA-A**	191	2735	6.98%
**HLA-B**	244	3455	7.06%
**HLA-C**	146	2259	6.46%
**HLA-DRB1**	143	1569	9.11%
**HLA-DQB1**	47	594	7.91%
**Total**	771	10612	7.27%

^a:^ data from http://www.ebi.ac.uk/ipd/imgt/hla/stats.html, 3.16 Release, April, 2014.

The three most common alleles for each of the five loci were as follows: A*11:01 (21.143%), A*24:02 (15.569%), and A*02:01 (12.290%); B*46:01 (10.221%), B*40:01 (9.956%) and B*58:01 (5.869%); C*01:02 (15.558%), C*07:02 (15.152%) and C*03:04 (9.957%); DRB1*09:01 (14.317%), DRB1*15:01 (11.652%) and DRB1*07:01 (8.911%); and DQB1*03:01 (21.094%), DQB1*03:03 (15.703%) and DQB1*06:01(10.183%) (S1–S5 Tables). These results are similar to those from our previous small-scale data reported by Hei et al. [17].

Hardy-Weinberg exact tests were performed on each of the five loci. The corresponding p values were 0.1948, 0.1412, 0.3952, 0.0807, and 0.0036 for the *HLA-A*, *HLA-C*, *HLA-B*, *HLA-DRB1*, and *HLA-DQB1* genes, respectively. The only significant deviation was observed for the DQB1 locus (Table 2).

**Table 2:** Hardy-Weinberg equilibrium tests for the HLA-A, -C, -B, -DRB1, and -DQB1 loci in 169,995 Chinese individuals.

Locus	HWE test (P value)	heterozygosity	
		Observed	Expected
**A**	0.1948	0.8772	0.9652
**C**	0.1412	0.9003	0.9849
**B**	0.3952	0.9433	0.9544
**DRB1**	0.0807	0.9230	0.9681
**DQB1**	0.0036	0.8808	0.9649

Observed heterozygosity = observed frequencies of heterozygotes;

Expected heterozygosity = expected frequencies of heterzygotes.

P value derived from Hardy-weinberg exact test.

### Common and well-documented alleles (CWD)

The alleles observed in our study were segregated into three categories based on the criteria described by Mack et al. [37]: common alleles (C), well-documented alleles (WD), and rare alleles (R). Totals of 41.4%, 54.5%, 46.6%, 52.4%, and 61.7% of the *HLA-A*, *-B*, *-C*, *-DRB1*, and-*DQB1* alleles were CWD for the Chinese population (Table 3). Among these CWD alleles, 25, 54, 25, 36, and 16 of the *HLA –A*, *-B*, *-C*, *-DRB1*, and-*DQB1* alleles, respectively, were common alleles with frequencies greater than 0.001 (Table 3). Totals of 54, 79, 43, 39 and 13 of the *HLA –A*, *-B*, *-C*, *-DRB1*, and -*DQB1* alleles, respectively, were well-documented alleles that were observed in at least five independent, unrelated individuals or in at least three independent, unrelated individuals sharing a haplotype (Table 3). Totals of 112, 111, 78, 68, and 18 for the *HLA-A*, *-B*, *-C*, *-DRB1*, and-*DQB1* alleles, respectively, were rare alleles. In the present study, less than 0.2% of the samples contained rare alleles, and more than 99.8% of the individuals carried alleles assigned to CWD category.

**Table 3:** Numbers of CWD alleles in 169,995 Chinese individuals.

Locus	Number of CWD alleles observed in this study ^a^			number of HLA alleles	Ratio ^b^
	C	WD	Total		
A	25	54	79	191	41.4%
B	54	79	133	244	54.5%
C	25	43	68	146	46.6%
DRB1	36	39	75	143	52.4%
DQB1	16	13	29	47	61.7%
TOTAL	156	228	384	771	49.8%

^a:^ according to the criteria published in Tissue Antigens, 2013, 81, 194–203.

^b:^ the ratio of the number of CWD alleles to the number of each of the HLA alleles observed in this study

C: common alleles;

WD: well-documented alleles.

### Haplotype frequencies and linkage disequilibria

The haplotypes HLA-A-C, HLA-C-B, HLA-DRB1-DQB1, HLA-A-C-B, HLA-A-B-DRB1, HLA-A-C-B-DRB1 and HLA-A-C-B-DRB1-DQB1 were estimated with the EM algorithm using the Arlequin program. Only the haplotypes with frequencies greater than 1‰ are included in S6–S9 Tables. The likelihood ratios test for the linkage disequilibrium of HLA-A-C, C-B and DRB1-DQB1 revealed that the vast majority of the pairwise associations were statistically significant (P <0.001; S6 Table).

The most frequent HLA-A-C-B haplotypes were A*02:07-C*01:02-B*46:01 (54.4‰) followed by A*30:01-C*06:02-B*13:02 (45.4‰), A*33:03-C*03:02-B*58:01 (44.3‰), A*11:01-C*07:02-B* 40:01 (22.9‰), and A*11:01-C*08:01-B*15:02 (22.0‰).

The most common HLA-A-C-B-DRB1-DQB1 haplotypes (S9 Table) with frequencies greater than 10‰ were A*30:01-C*06:02-B*13:02-DRB1*07:01-DQB1*02:02 (37.0‰), A*02:07-C*01:02-B*46:01-DRB1*09:01-DQB1*03:03 (24.6‰), A*33:03-C*03:02-B*58:01-DRB1*03:01-DQB1*02:01 (24.0‰), A*11:01-C*08:01-B*15:02-DRB1*12:02-DQB1*03:01 (11.3‰) and A*33:03-C*03:02-B*58:01-DRB1*13:02-DQB1*06:09 (10.6‰). These five most common HLA-A-C-B-DRB1-DQB1 haplotypes overlapped with the five most frequent HLA-A-B-DRB1 and HLA-A-C-B-DRB1 haplotypes with frequencies greater than 10‰.

### Regional distributions of several allelic categories

**Fig 1:**
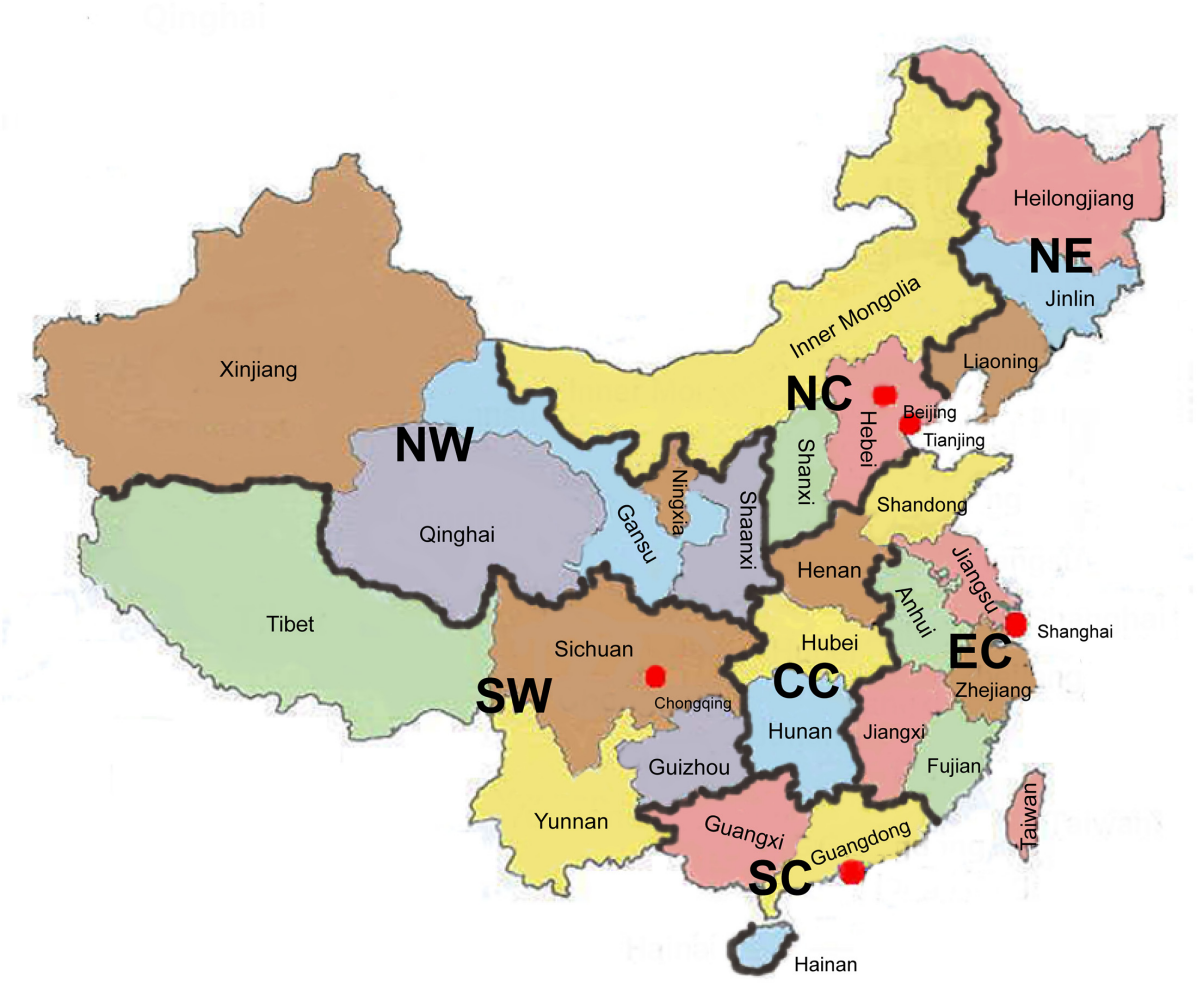
Geographic regions of China. Northeast China (NE), North China (NC), Northwest China (NW), East China (EC), Central China (CC), South China (SC), and Southwest China (SW). Note: Taiwan, Hong Kong and Macau were not included in this study.

China can be traditionally divided into seven geographical regions, i.e., Northeast China (NE), North China (NC), Northwest China (NW), East China (EC), Central China (CC), South China (SC), and Southwest China (SW), from the north to the south (Fig 1). To identify the characteristic distributions of the HLA diversities, regional partitioning was performed based on the seven geographical regions.

To examine the regional differences in HLA allelic variations, all of the alleles were divided into several categories that included CWD alleles, rare alleles, not-observed alleles, shared-alleles (in all regions) and unique alleles (in only one region) based on observed population allele frequencies for *HLA-A*, *-B*, *-C*, *-DRB1*, and-*DQB1*. Table 4 shows the numbers of alleles in several categories observed in each of the seven regions. The CWD type alleles accounted for 21% to 28% for *HLA-A*, 32% to 42% for *HLA-B*, 23% to 32% for *HLA-C*, 29% to 39% for *HLA-DRB1* and 40% to 55% for *HLA-DQB1*. Among the seven regions, the greatest number of CWD alleles was observed among individuals from East China; however, regarding the average numbers of CWD alleles per 1000 individuals, East China's contribution was the smallest. The numbers of shared alleles in all of the seven regions were 47 (24.6%) for *HLA-A*, 90 (36.9%) for *HLA-B*, 42 (28.8%) for *HLA-C*, 51 (35.7%) for *HLA-DRB1* and 23 (48.9%) for *HLA-DQB1*. Moreover, these unique alleles observed in each of the seven regions were extreme rare; they were present in fewer, 0.04% of the observed individuals.

**Table 4:** Numbers of HLA alleles according to several categories observed in the seven geographic regions.

HLA allele	NE			NC			NW			EC			CC			SC			SW		
categories	allele acount	alleles %	sample%	allele acount	alleles %	sample%	allele acount	alleles %	sample%	allele acount	alleles %	sample%	allele acount	alleles %	sample%	allele acount	alleles %	sample%	allele acount	alleles %	sample%
**HLA-A**																					
CWD	43	22.5	99.78	50	26.2	99.87	42	22.0	99.79	53	27.7	99.91	45	23.6	99.84	40	20.9	99.81	44	23.0	99.85
Rare	34	17.8	0.22	47	24.6	0.13	33	17.3	0.21	57	29.8	0.09	48	25.1	0.16	45	23.6	0.19	36	18.8	0.15
Not observed	114	59.7	0	94	49.2	0	116	60.7	0	81	42.4	0	98	51.3	0	106	55.5	0	111	58.1	0
TOTAL	191	100.0	100.0	191	100.0	100.0	191	100.0	100.0	191	100.0	100.0	191	100.0	100.0	191	100.0	100.0	191	100.0	100.0
Shared in all regions	47	24.6	99.79	47	24.6	99.81	47	24.6	99.84	47	24.6	99.87	47	24.6	99.83	47	24.6	99.84	47	24.6	99.81
Unique in one region	4	2.1	0.02	16	8.4	0.03	7	3.7	0.02	16	8.4	0.02	13	6.8	0.03	11	5.8	0.03	10	5.2	0.02
**HLA-B**																					
CWD	77	31.6	99.63	96	39.3	99.83	83	34.0	99.69	102	41.8	99.88	88	36.1	99.82	80	32.8	99.83	93	38.1	99.84
Rare	49	20.1	0.37	52	21.3	0.17	53	21.7	0.31	73	29.9	0.12	59	24.2	0.18	34	13.9	0.17	35	14.3	0.16
Not observed	118	48.4	0	96	39.3	0	108	44.3	0	69	28.3	0	97	39.8	0	130	53.3	0	116	47.5	0
TOTAL	244	100.0	100.0	244	100.0	100.0	244	100.0	100.0	244	100.0	100.0	244	100.0	100.0	244	100.0	100.0	244	100.0	100.0
Shared in all regions	90	36.9	99.67	90	36.9	99.68	90	36.9	99.58	90	36.9	99.72	90	36.9	99.79	90	36.9	99.84	90	36.9	99.66
Unique in one region	4	1.6	0.02	18	7.4	0.04	7	2.9	0.02	28	11.5	0.03	14	5.7	0.03	7	2.9	0.02	5	2.0	0.03
**HLA-C**																					
CWD	34	23.3	99.79	41	28.1	99.88	36	24.66	99.82	47	32.2	99.93	42	28.8	99.90	38	26.0	99.87	37	25.3	99.84
Rare	29	19.9	0.21	37	25.3	0.12	36	24.66	0.18	47	32.2	0.07	32	21.9	0.10	36	24.7	0.13	40	27.4	0.16
Not observed	83	56.8	0	68	46.6	0	74	50.68	0	52	35.6	0	72	49.3	0	72	49.3	0	69	47.3	0
TOTAL	146	100.0	100.0	146	100.0	100.0	146	100.0	100.0	146	100.0	100.0	146	100.0	100.0	146	100.0	100.0	146	100.0	100.0
shared in all regions	42	28.8	99.86	42	28.8	99.87	42	28.8	99.84	42	28.8	99.87	42	28.8	99.83	42	28.8	99.77	42	28.8	99.83
Unique in one region	3	2.1	0.01	6	4.1	0.01	7	4.8	0.02	13	8.9	0.01	7	4.8	0.02	7	4.8	0.03	6	4.1	0.01
**HLA-DRB1**																					
CWD	47	32.9	99.83	55	38.5	99.94	49	34.3	99.86	56	39.2	99.94	51	35.7	99.88	42	29.4	99.85	51	35.7	99.90
Rare	21	14.7	0.17	23	16.1	0.06	23	16.1	0.14	36	25.2	0.06	41	28.7	0.12	31	21.7	0.15	28	19.6	0.10
Not observed	75	52.4	0	65	45.5	0	71	49.7	0	51	35.7	0	51	35.7	0	70	49.0	0	64	44.8	0
TOTAL	143	100.0	100.0	143	100.0	100.0	143	100.0	100.0	143	100.0	100.0	143	100.0	100.0	143	100.0	100.0	143	100.0	100.0
Shared in all regions	51	35.7	99.84	51	35.7	99.85	51	35.7	99.84	51	35.7	99.89	51	35.7	99.86	51	35.7	99.91	51	35.7	99.79
Unique in one region	3	2.1	0.01	5	3.5	0.01	4	2.8	0.01	12	8.4	0.02	12	8.4	0.02	6	4.2	0.02	8	5.6	0.03
**HLA-DQB1**																					
CWD	20	42.6	99.94	21	44.7	99.98	19	40.4	99.94	26	55.3	99.99	23	48.9	99.97	19	40.4	99.97	21	44.7	99.97
Rare	8	17.0	0.06	7	14.9	0.02	8	17.0	0.06	8	17.0	0.01	10	21.3	0.03	10	21.3	0.03	9	19.1	0.03
Not observed	19	40.4	0	19	40.4	0	20	42.6	0	13	27.7	0	14	29.8	0	18	38.3	0	17	36.2	0
TOTAL	47	100.0	100.0	47	100.0	100.0	47	100.0	100.0	47	100.0	100.0	47	100.0	100.0	47	100.0	100.0	47	100.0	100.0
Shared in all regions	23	48.9	99.98	23	48.9	99.99	23	48.9	99.97	23	48.9	99.97	23	48.9	99.96	23	48.9	99.98	23	48.9	99.98
Unique in one region	1	2.1	0.004	1	2.1	0.002	1	2.1	0.003	2	4.3	0.002	4	8.5	0.008	3	6.4	0.008	1	2.1	0.002

CWD: common and well-documented allele; Rare: rare allele;

Not observed: alleles that were absent in one region;

Shared in all regions: alleles that were observed in all regions;

Unique in one region: alleles that were only observed in one region.

### Characteristics and regional differences in five-locus haplotypes

**Fig 2:**
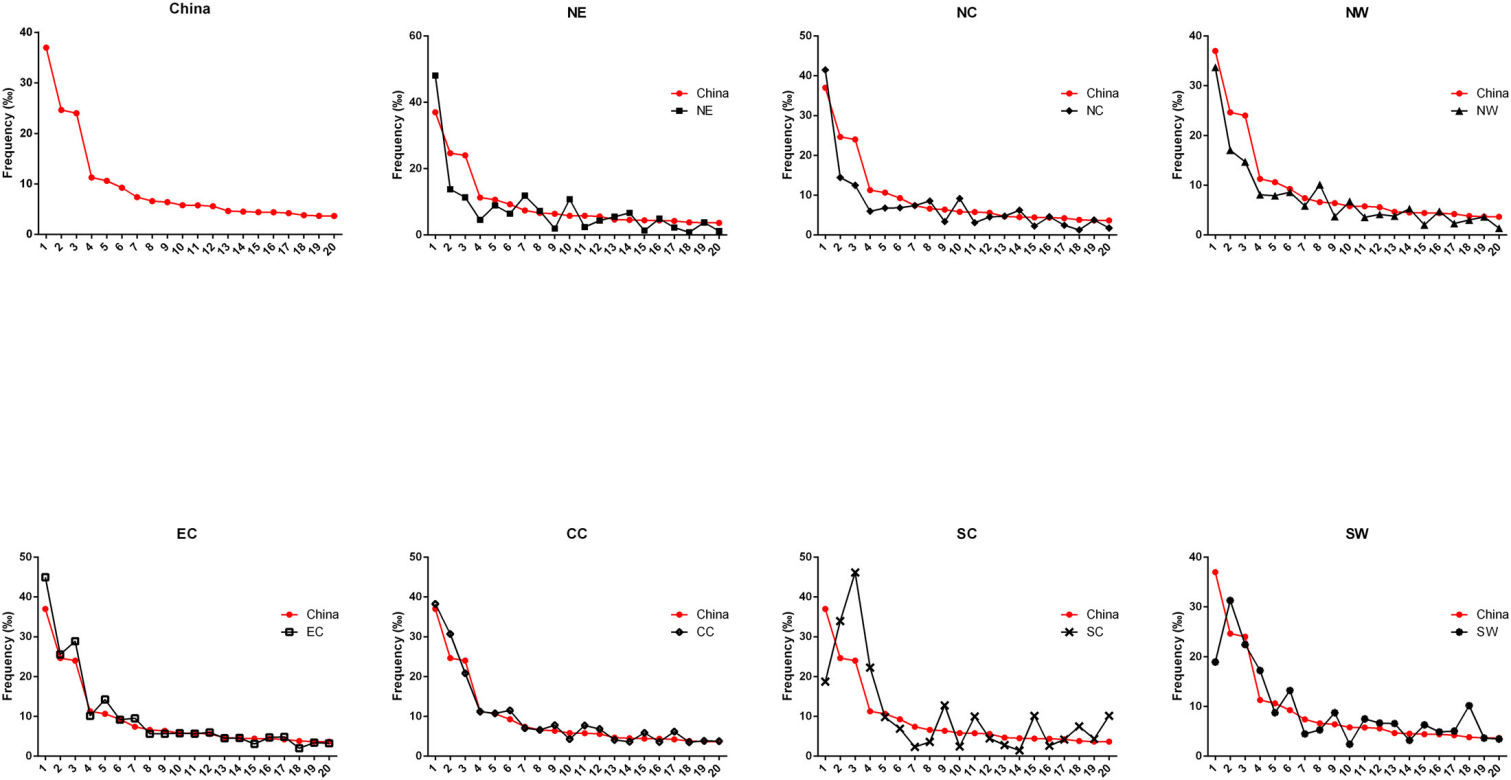
Distributions of the 20 most common HLA five-locus haplotypes in the seven geographic regions of China. (A) China; (B) Northeast China (NE); (C) North China (NC); (D) Northwest China (NW); (E) East China (EC); (F) Central China (CC); (G) South China (SC); (H) Southwest China (SW).

To further characterize the regional differences in HLA variation, we compared the distributions of the A-C-B-DRB1-DQB1 haplotypes in seven regions in detail. First, we compared the top 20 most common haplotypes in each region (Table 5), and a graphical representation of this comparison is shown in Fig 2. Second, in order to confirm the regionally specific haplotypes, we analyzed the frequencies of the 100 most common haplotypes among the regions. Regionally specific haplotypes were defined as haplotypes with frequencies in one region that were at least two times higher than those in all other regions.

**Table 5:** Occurrences of the first 20 HLA A-C-B-DRB1-DQB1 frequency-ranked haplotypes for each region in each of the other geographic regions of China.

Rank	Haplotype					China		NE		NC		NW		EC		CC		SC		SW	
	A	C	B	DRB1	DQB1	Rank	Freq.(‰)	Rank	Freq.(‰)	Rank	Freq.(‰)	Rank	Freq.(‰)	Rank	Freq.(‰)	Rank	Freq.(‰)	Rank	Freq.(‰)	Rank	Freq.(‰)
**China**																					
	30:01	06:02	13:02	07:01	02:02	**1**	37.00	1	48.06	1	41.47	1	33.69	1	44.96	1	38.26	4	18.72	3	18.93
	02:07	01:02	46:01	09:01	03:03	**2**	24.64	2	13.78	2	14.43	2	17.05	3	25.56	2	30.70	2	33.94	1	31.30
	33:03	03:02	58:01	03:01	02:01	**3**	24.00	4	11.33	3	12.47	3	14.70	2	28.88	3	20.83	1	46.15	2	22.42
	11:01	08:01	15:02	12:02	03:01	**4**	11.27	13	4.57	10	5.95	6	8.08	5	10.11	5	11.19	3	22.25	4	17.22
	33:03	03:02	58:01	13:02	06:09	**5**	10.62	6	8.91	8	6.74	7	7.90	4	14.26	6	10.76	9	9.81	8	8.72
	02:07	01:02	46:01	08:03	06:01	**6**	9.25	9	6.41	7	6.80	5	8.59	7	9.16	4	11.48	12	6.89	5	13.21
	33:03	14:03	44:03	13:02	06:04	**7**	7.39	3	11.82	6	7.38	9	5.82	6	9.52	9	7.04	40	2.29	19	4.45
	01:01	06:02	37:01	10:01	05:01	**8**	6.60	7	7.25	5	8.53	4	10.10	12	5.62	11	6.58	21	3.56	15	5.27
	11:01	03:04	13:01	15:01	06:01	**9**	6.38	37	1.93	22	3.36	18	3.71	11	5.63	7	7.78	5	12.75	7	8.74
	02:01	03:04	13:01	12:02	03:01	**10**	5.79	5	10.75	4	9.15	8	6.73	9	5.75	15	4.26	33	2.44	37	2.37
	11:01	01:02	46:01	09:01	03:03	**11**	5.76	28	2.37	26	3.06	21	3.55	10	5.64	8	7.69	8	9.97	10	7.48
	11:01	04:01	15:01	04:06	03:02	**12**	5.58	15	4.31	14	4.54	16	4.14	8	5.97	10	6.81	14	4.46	11	6.65
	33:03	07:06	44:03	07:01	02:02	**13**	4.65	10	5.46	12	4.72	17	3.78	17	4.49	16	4.07	26	2.79	13	6.54
	01:01	06:02	57:01	07:01	03:03	**14**	4.50	8	6.64	9	6.20	10	5.27	16	4.56	20	3.61	77	1.42	30	3.20
	11:01	08:01	15:02	15:01	06:01	**15**	4.40	76	1.32	36	2.24	41	2.03	25	3.06	13	5.86	6	10.12	14	6.30
	24:02	01:02	54:01	04:05	04:01	**16**	4.38	11	4.90	13	4.60	12	4.76	14	4.67	21	3.57	31	2.59	18	4.86
	11:01	07:02	40:01	08:03	06:01	**17**	4.21	30	2.24	32	2.44	33	2.30	13	4.85	12	6.14	17	4.18	17	5.03
	02:07	01:02	46:01	14:54	05:02	**18**	3.80	149	0.78	78	1.26	26	3.00	40	1.97	22	3.51	11	7.49	6	10.14
	11:01	03:04	13:01	12:02	03:01	**19**	3.67	18	3.75	18	3.76	19	3.61	21	3.37	17	3.84	15	4.34	24	3.63
	02:03	07:02	38:02	16:02	05:02	**20**	3.64	93	1.15	51	1.73	64	1.35	24	3.22	18	3.78	7	10.11	26	3.45
**NE**																					
	30:01	06:02	13:02	07:01	02:02	1	37.00	**1**	48.06	1	41.47	1	33.69	1	44.96	1	38.26	4	18.72	3	18.93
	02:07	01:02	46:01	09:01	03:03	2	24.64	**2**	13.78	2	14.43	2	17.05	3	25.56	2	30.70	2	33.94	1	31.30
	33:03	14:03	44:03	13:02	06:04	7	7.39	**3**	11.82	6	7.38	9	5.82	6	9.52	9	7.04	40	2.29	19	4.45
	33:03	03:02	58:01	03:01	02:01	3	24.00	**4**	11.33	3	12.47	3	14.70	2	28.88	3	20.83	1	46.15	2	22.42
	02:01	03:04	13:01	12:02	03:01	10	5.79	**5**	10.75	4	9.15	8	6.73	9	5.75	15	4.26	33	2.44	37	2.37
	33:03	03:02	58:01	13:02	06:09	5	10.62	**6**	8.91	8	6.74	7	7.90	4	14.26	6	10.76	9	9.81	8	8.72
	01:01	06:02	37:01	10:01	05:01	8	6.60	**7**	7.25	5	8.53	4	10.10	12	5.62	11	6.58	21	3.56	15	5.27
	01:01	06:02	57:01	07:01	03:03	14	4.50	**8**	6.64	9	6.20	10	5.27	16	4.56	20	3.61	77	1.42	30	3.20
	02:07	01:02	46:01	08:03	06:01	6	9.25	**9**	6.41	7	6.80	5	8.59	7	9.16	4	11.48	12	6.89	5	13.21
	33:03	07:06	44:03	07:01	02:02	13	4.65	**10**	5.46	12	4.72	17	3.78	17	4.49	16	4.07	26	2.79	13	6.54
	24:02	01:02	54:01	04:05	04:01	16	4.38	**11**	4.90	13	4.60	12	4.76	14	4.67	21	3.57	31	2.59	18	4.86
	02:01	03:03	15:11	09:01	03:03	22	3.55	**12**	4.75	15	4.21	15	4.25	18	4.02	24	3.25	72	1.45	43	2.22
	11:01	08:01	15:02	12:02	03:01	4	11.27	**13**	4.57	10	5.95	6	8.08	5	10.11	5	11.19	3	22.25	4	17.22
	03:01	07:02	07:02	15:01	06:02	28	2.84	**14**	4.45	11	5.20	25	3.13	32	2.49	34	2.32	133	0.93	100	1.04
	11:01	04:01	15:01	04:06	03:02	12	5.58	**15**	4.31	14	4.54	16	4.14	8	5.97	10	6.81	14	4.46	11	6.65
	11:01	12:02	52:01	15:02	06:01	30	2.57	**16**	4.09	16	3.96	13	4.70	36	2.14	33	2.46	104	1.11	84	1.19
	32:01	12:02	52:01	15:02	06:01	23	3.18	**17**	3.82	17	3.83	14	4.40	23	3.31	26	3.07	112	1.06	58	1.65
	11:01	03:04	13:01	12:02	03:01	19	3.67	**18**	3.75	18	3.76	19	3.61	21	3.37	17	3.84	15	4.34	24	3.63
	03:01	05:01	44:02	13:01	06:03	32	2.43	**19**	3.75	23	3.29	24	3.16	60	1.60	49	1.83	205	0.64	23	3.78
	11:01	14:02	51:01	09:01	03:03	25	3.14	**20**	3.70	31	2.44	20	3.60	20	3.38	23	3.38	35	2.40	28	3.31
**NC**																					
	30:01	06:02	13:02	07:01	02:02	1	37.00	1	48.06	**1**	41.47	1	33.69	1	44.96	1	38.26	4	18.72	3	18.93
	02:07	01:02	46:01	09:01	03:03	2	24.64	2	13.78	**2**	14.43	2	17.05	3	25.56	2	30.70	2	33.94	1	31.30
	33:03	03:02	58:01	03:01	02:01	3	24.00	4	11.33	**3**	12.47	3	14.70	2	28.88	3	20.83	1	46.15	2	22.42
	02:01	03:04	13:01	12:02	03:01	10	5.79	5	10.75	**4**	9.15	8	6.73	9	5.75	15	4.26	33	2.44	37	2.37
	01:01	06:02	37:01	10:01	05:01	8	6.60	7	7.25	**5**	8.53	4	10.10	12	5.62	11	6.58	21	3.56	15	5.27
	33:03	14:03	44:03	13:02	06:04	7	7.39	3	11.82	**6**	7.38	9	5.82	6	9.52	9	7.04	40	2.29	19	4.45
	02:07	01:02	46:01	08:03	06:01	6	9.25	9	6.41	**7**	6.80	5	8.59	7	9.16	4	11.48	12	6.89	5	13.21
	33:03	03:02	58:01	13:02	06:09	5	10.62	6	8.91	**8**	6.74	7	7.90	4	14.26	6	10.76	9	9.81	8	8.72
	01:01	06:02	57:01	07:01	03:03	14	4.50	8	6.64	**9**	6.20	10	5.27	16	4.56	20	3.61	77	1.42	30	3.20
	11:01	08:01	15:02	12:02	03:01	4	11.27	13	4.57	**10**	5.95	6	8.08	5	10.11	5	11.19	3	22.25	4	17.22
	03:01	07:02	07:02	15:01	06:02	28	2.84	14	4.45	**11**	5.20	25	3.13	32	2.49	34	2.32	133	0.93	100	1.04
	33:03	07:06	44:03	07:01	02:02	13	4.65	10	5.46	**12**	4.72	17	3.78	17	4.49	16	4.07	26	2.79	13	6.54
	24:02	01:02	54:01	04:05	04:01	16	4.38	11	4.90	**13**	4.60	12	4.76	14	4.67	21	3.57	31	2.59	18	4.86
	11:01	04:01	15:01	04:06	03:02	12	5.58	15	4.31	**14**	4.54	16	4.14	8	5.97	10	6.81	14	4.46	11	6.65
	02:01	03:03	15:11	09:01	03:03	22	3.55	12	4.75	**15**	4.21	15	4.25	18	4.02	24	3.25	72	1.45	43	2.22
	11:01	12:02	52:01	15:02	06:01	30	2.57	16	4.09	**16**	3.96	13	4.70	36	2.14	33	2.46	104	1.11	84	1.19
	32:01	12:02	52:01	15:02	06:01	23	3.18	17	3.82	**17**	3.83	14	4.40	23	3.31	26	3.07	112	1.06	58	1.65
	11:01	03:04	13:01	12:02	03:01	19	3.67	18	3.75	**18**	3.76	19	3.61	21	3.37	17	3.84	15	4.34	24	3.63
	32:01	04:01	44:03	07:01	02:02	37	2.23	27	2.48	**19**	3.70	27	2.92	49	1.77	28	2.95	250	0.51	119	0.90
	24:02	14:02	51:01	09:01	03:03	26	3.13	25	2.62	**20**	3.57	23	3.22	19	3.48	25	3.20	42	2.25	35	2.66
**NW**																					
	30:01	06:02	13:02	07:01	02:02	1	37.00	1	48.06	1	41.47	**1**	33.69	1	44.96	1	38.26	4	18.72	3	18.93
	02:07	01:02	46:01	09:01	03:03	2	24.64	2	13.78	2	14.43	**2**	17.05	3	25.56	2	30.70	2	33.94	1	31.30
	33:03	03:02	58:01	03:01	02:01	3	24.00	4	11.33	3	12.47	**3**	14.70	2	28.88	3	20.83	1	46.15	2	22.42
	01:01	06:02	37:01	10:01	05:01	8	6.60	7	7.25	5	8.53	**4**	10.10	12	5.62	11	6.58	21	3.56	15	5.27
	02:07	01:02	46:01	08:03	06:01	6	9.25	9	6.41	7	6.80	**5**	8.59	7	9.16	4	11.48	12	6.89	5	13.21
	11:01	08:01	15:02	12:02	03:01	4	11.27	13	4.57	10	5.95	**6**	8.08	5	10.11	5	11.19	3	22.25	4	17.22
	33:03	03:02	58:01	13:02	06:09	5	10.62	6	8.91	8	6.74	**7**	7.90	4	14.26	6	10.76	9	9.81	8	8.72
	02:01	03:04	13:01	12:02	03:01	10	5.79	5	10.75	4	9.15	**8**	6.73	9	5.75	15	4.26	33	2.44	37	2.37
	33:03	14:03	44:03	13:02	06:04	7	7.39	3	11.82	6	7.38	**9**	5.82	6	9.52	9	7.04	40	2.29	19	4.45
	01:01	06:02	57:01	07:01	03:03	14	4.50	8	6.64	9	6.20	**10**	5.27	16	4.56	20	3.61	77	1.42	30	3.20
	11:01	07:02	07:02	01:01	05:01	27	2.89	45	1.83	30	2.46	**11**	4.92	46	1.83	30	2.63	107	1.08	12	6.62
	24:02	01:02	54:01	04:05	04:01	16	4.38	11	4.90	13	4.60	**12**	4.76	14	4.67	21	3.57	31	2.59	18	4.86
	11:01	12:02	52:01	15:02	06:01	30	2.57	16	4.09	16	3.96	**13**	4.70	36	2.14	33	2.46	104	1.11	84	1.19
	32:01	12:02	52:01	15:02	06:01	23	3.18	17	3.82	17	3.83	**14**	4.40	23	3.31	26	3.07	112	1.06	58	1.65
	02:01	03:03	15:11	09:01	03:03	22	3.55	12	4.75	15	4.21	**15**	4.25	18	4.02	24	3.25	72	1.45	43	2.22
	11:01	04:01	15:01	04:06	03:02	12	5.58	15	4.31	14	4.54	**16**	4.14	8	5.97	10	6.81	14	4.46	11	6.65
	33:03	07:06	44:03	07:01	02:02	13	4.65	10	5.46	12	4.72	**17**	3.78	17	4.49	16	4.07	26	2.79	13	6.54
	11:01	03:04	13:01	15:01	06:01	9	6.38	37	1.93	22	3.36	**18**	3.71	11	5.63	7	7.78	5	12.75	7	8.74
	11:01	03:04	13:01	12:02	03:01	19	3.67	18	3.75	18	3.76	**19**	3.61	21	3.37	17	3.84	15	4.34	24	3.63
	11:01	14:02	51:01	09:01	03:03	25	3.14	20	3.70	31	2.44	**20**	3.60	20	3.38	23	3.38	35	2.40	28	3.31
**EC**																					
	30:01	06:02	13:02	07:01	02:02	1	37.00	1	48.06	1	41.47	1	33.69	**1**	44.96	1	38.26	4	18.72	3	18.93
	33:03	03:02	58:01	03:01	02:01	3	24.00	4	11.33	3	12.47	3	14.70	**2**	28.88	3	20.83	1	46.15	2	22.42
	02:07	01:02	46:01	09:01	03:03	2	24.64	2	13.78	2	14.43	2	17.05	**3**	25.56	2	30.70	2	33.94	1	31.30
	33:03	03:02	58:01	13:02	06:09	5	10.62	6	8.91	8	6.74	7	7.90	**4**	14.26	6	10.76	9	9.81	8	8.72
	11:01	08:01	15:02	12:02	03:01	4	11.27	13	4.57	10	5.95	6	8.08	**5**	10.11	5	11.19	3	22.25	4	17.22
	33:03	14:03	44:03	13:02	06:04	7	7.39	3	11.82	6	7.38	9	5.82	**6**	9.52	9	7.04	40	2.29	19	4.45
	02:07	01:02	46:01	08:03	06:01	6	9.25	9	6.41	7	6.80	5	8.59	**7**	9.16	4	11.48	12	6.89	5	13.21
	11:01	04:01	15:01	04:06	03:02	12	5.58	15	4.31	14	4.54	16	4.14	**8**	5.97	10	6.81	14	4.46	11	6.65
	02:01	03:04	13:01	12:02	03:01	10	5.79	5	10.75	4	9.15	8	6.73	**9**	5.75	15	4.26	33	2.44	37	2.37
	11:01	01:02	46:01	09:01	03:03	11	5.76	28	2.37	26	3.06	21	3.55	**10**	5.64	8	7.69	8	9.97	10	7.48
	11:01	03:04	13:01	15:01	06:01	9	6.38	37	1.93	22	3.36	18	3.71	**11**	5.63	7	7.78	5	12.75	7	8.74
	01:01	06:02	37:01	10:01	05:01	8	6.60	7	7.25	5	8.53	4	10.10	**12**	5.62	11	6.58	21	3.56	15	5.27
	11:01	07:02	40:01	08:03	06:01	17	4.21	30	2.24	32	2.44	33	2.30	**13**	4.85	12	6.14	17	4.18	17	5.03
	24:02	01:02	54:01	04:05	04:01	16	4.38	11	4.90	13	4.60	12	4.76	**14**	4.67	21	3.57	31	2.59	18	4.86
	11:01	07:02	40:01	09:01	03:03	21	3.59	46	1.83	44	1.95	43	2.01	**15**	4.58	14	4.95	18	4.01	25	3.63
	01:01	06:02	57:01	07:01	03:03	14	4.50	8	6.64	9	6.20	10	5.27	**16**	4.56	20	3.61	77	1.42	30	3.20
	33:03	07:06	44:03	07:01	02:02	13	4.65	10	5.46	12	4.72	17	3.78	**17**	4.49	16	4.07	26	2.79	13	6.54
	02:01	03:03	15:11	09:01	03:03	22	3.55	12	4.75	15	4.21	15	4.25	**18**	4.02	24	3.25	72	1.45	43	2.22
	24:02	14:02	51:01	09:01	03:03	26	3.13	25	2.62	20	3.57	23	3.22	**19**	3.48	25	3.20	42	2.25	35	2.66
	11:01	14:02	51:01	09:01	03:03	25	3.14	20	3.70	31	2.44	20	3.60	**20**	3.38	23	3.38	35	2.40	28	3.31
**CC**																					
	30:01	06:02	13:02	07:01	02:02	1	37.00	1	48.06	1	41.47	1	33.69	1	44.96	**1**	38.26	4	18.72	3	18.93
	02:07	01:02	46:01	09:01	03:03	2	24.64	2	13.78	2	14.43	2	17.05	3	25.56	**2**	30.70	2	33.94	1	31.30
	33:03	03:02	58:01	03:01	02:01	3	24.00	4	11.33	3	12.47	3	14.70	2	28.88	**3**	20.83	1	46.15	2	22.42
	02:07	01:02	46:01	08:03	06:01	6	9.25	9	6.41	7	6.80	5	8.59	7	9.16	**4**	11.48	12	6.89	5	13.21
	11:01	08:01	15:02	12:02	03:01	4	11.27	13	4.57	10	5.95	6	8.08	5	10.11	**5**	11.19	3	22.25	4	17.22
	33:03	03:02	58:01	13:02	06:09	5	10.62	6	8.91	8	6.74	7	7.90	4	14.26	**6**	10.76	9	9.81	8	8.72
	11:01	03:04	13:01	15:01	06:01	9	6.38	37	1.93	22	3.36	18	3.71	11	5.63	**7**	7.78	5	12.75	7	8.74
	11:01	01:02	46:01	09:01	03:03	11	5.76	28	2.37	26	3.06	21	3.55	10	5.64	**8**	7.69	8	9.97	10	7.48
	33:03	14:03	44:03	13:02	06:04	7	7.39	3	11.82	6	7.38	9	5.82	6	9.52	**9**	7.04	40	2.29	19	4.45
	11:01	04:01	15:01	04:06	03:02	12	5.58	15	4.31	14	4.54	16	4.14	8	5.97	**10**	6.81	14	4.46	11	6.65
	01:01	06:02	37:01	10:01	05:01	8	6.60	7	7.25	5	8.53	4	10.10	12	5.62	**11**	6.58	21	3.56	15	5.27
	11:01	07:02	40:01	08:03	06:01	17	4.21	30	2.24	32	2.44	33	2.30	13	4.85	**12**	6.14	17	4.18	17	5.03
	11:01	08:01	15:02	15:01	06:01	15	4.40	76	1.32	36	2.24	41	2.03	25	3.06	**13**	5.86	6	10.12	14	6.30
	11:01	07:02	40:01	09:01	03:03	21	3.59	46	1.83	44	1.95	43	2.01	15	4.58	**14**	4.95	18	4.01	25	3.63
	02:01	03:04	13:01	12:02	03:01	10	5.79	5	10.75	4	9.15	8	6.73	9	5.75	**15**	4.26	33	2.44	37	2.37
	33:03	07:06	44:03	07:01	02:02	13	4.65	10	5.46	12	4.72	17	3.78	17	4.49	**16**	4.07	26	2.79	13	6.54
	11:01	03:04	13:01	12:02	03:01	19	3.67	18	3.75	18	3.76	19	3.61	21	3.37	**17**	3.84	15	4.34	24	3.63
	02:03	07:02	38:02	16:02	05:02	20	3.64	93	1.15	51	1.73	64	1.35	24	3.22	**18**	3.78	7	10.11	26	3.45
	24:02	01:02	46:01	09:01	03:03	24	3.14	53	1.62	39	1.99	28	2.66	22	3.32	**19**	3.61	13	4.52	22	3.98
	01:01	06:02	57:01	07:01	03:03	14	4.50	8	6.64	9	6.20	10	5.27	16	4.56	**20**	3.61	77	1.42	30	3.20
**SC**																					
	33:03	03:02	58:01	03:01	02:01	3	24.00	4	11.33	3	12.47	3	14.70	2	28.88	3	20.83	**1**	46.15	2	22.42
	02:07	01:02	46:01	09:01	03:03	2	24.64	2	13.78	2	14.43	2	17.05	3	25.56	2	30.70	**2**	33.94	1	31.30
	11:01	08:01	15:02	12:02	03:01	4	11.27	13	4.57	10	5.95	6	8.08	5	10.11	5	11.19	**3**	22.25	4	17.22
	30:01	06:02	13:02	07:01	02:02	1	37.00	1	48.06	1	41.47	1	33.69	1	44.96	1	38.26	**4**	18.72	3	18.93
	11:01	03:04	13:01	15:01	06:01	9	6.38	37	1.93	22	3.36	18	3.71	11	5.63	7	7.78	**5**	12.75	7	8.74
	11:01	08:01	15:02	15:01	06:01	15	4.40	76	1.32	36	2.24	41	2.03	25	3.06	13	5.86	**6**	10.12	14	6.30
	02:03	07:02	38:02	16:02	05:02	20	3.64	93	1.15	51	1.73	64	1.35	24	3.22	18	3.78	**7**	10.11	26	3.45
	11:01	01:02	46:01	09:01	03:03	11	5.76	28	2.37	26	3.06	21	3.55	10	5.64	8	7.69	**8**	9.97	10	7.48
	33:03	03:02	58:01	13:02	06:09	5	10.62	6	8.91	8	6.74	7	7.90	4	14.26	6	10.76	**9**	9.81	8	8.72
	11:01	03:04	13:01	16:02	05:02	31	2.45	215	0.59	133	0.83	78	1.23	41	1.91	39	2.17	**10**	7.71	34	2.88
	02:07	01:02	46:01	14:54	05:02	18	3.80	149	0.78	78	1.26	26	3.00	40	1.97	22	3.51	**11**	7.49	6	10.14
	02:07	01:02	46:01	08:03	06:01	6	9.25	9	6.41	7	6.80	5	8.59	7	9.16	4	11.48	**12**	6.89	5	13.21
	24:02	01:02	46:01	09:01	03:03	24	3.14	53	1.62	39	1.99	28	2.66	22	3.32	19	3.61	**13**	4.52	22	3.98
	11:01	04:01	15:01	04:06	03:02	12	5.58	15	4.31	14	4.54	16	4.14	8	5.97	10	6.81	**14**	4.46	11	6.65
	11:01	03:04	13:01	12:02	03:01	19	3.67	18	3.75	18	3.76	19	3.61	21	3.37	17	3.84	**15**	4.34	24	3.63
	29:01	15:05	07:05	10:01	05:01	52	1.65	148	0.78	122	0.88	66	1.34	92	1.13	88	1.19	**16**	4.27	39	2.32
	11:01	07:02	40:01	08:03	06:01	17	4.21	30	2.24	32	2.44	33	2.30	13	4.85	12	6.14	**17**	4.18	17	5.03
	11:01	07:02	40:01	09:01	03:03	21	3.59	46	1.83	44	1.95	43	2.01	15	4.58	14	4.95	**18**	4.01	25	3.63
	24:02	03:04	13:01	15:01	06:01	81	1.25	281	0.45	259	0.48	327	0.37	108	0.91	70	1.42	**19**	3.91	60	1.62
	11:01	03:02	58:01	03:01	02:01	42	1.83	169	0.72	94	1.16	106	0.97	38	2.05	44	1.98	**20**	3.77	65	1.54
**SW**																					
	02:07	01:02	46:01	09:01	03:03	2	24.64	2	13.78	2	14.43	2	17.05	3	25.56	2	30.70	2	33.94	**1**	31.30
	33:03	03:02	58:01	03:01	02:01	3	24.00	4	11.33	3	12.47	3	14.70	2	28.88	3	20.83	1	46.15	**2**	22.42
	30:01	06:02	13:02	07:01	02:02	1	37.00	1	48.06	1	41.47	1	33.69	1	44.96	1	38.26	4	18.72	**3**	18.93
	11:01	08:01	15:02	12:02	03:01	4	11.27	13	4.57	10	5.95	6	8.08	5	10.11	5	11.19	3	22.25	**4**	17.22
	02:07	01:02	46:01	08:03	06:01	6	9.25	9	6.41	7	6.80	5	8.59	7	9.16	4	11.48	12	6.89	**5**	13.21
	02:07	01:02	46:01	14:54	05:02	18	3.80	149	0.78	78	1.26	26	3.00	40	1.97	22	3.51	11	7.49	**6**	10.14
	11:01	03:04	13:01	15:01	06:01	9	6.38	37	1.93	22	3.36	18	3.71	11	5.63	7	7.78	5	12.75	**7**	8.74
	33:03	03:02	58:01	13:02	06:09	5	10.62	6	8.91	8	6.74	7	7.90	4	14.26	6	10.76	9	9.81	**8**	8.72
	24:02	04:03	15:25	12:02	03:01	68	1.35	338	0.39	403	0.34	244	0.47	377	0.36	643	0.20	181	0.72	**9**	7.90
	11:01	01:02	46:01	09:01	03:03	11	5.76	28	2.37	26	3.06	21	3.55	10	5.64	8	7.69	8	9.97	**10**	7.48
	11:01	04:01	15:01	04:06	03:02	12	5.58	15	4.31	14	4.54	16	4.14	8	5.97	10	6.81	14	4.46	**11**	6.65
	11:01	07:02	07:02	01:01	05:01	27	2.89	45	1.83	30	2.46	11	4.92	46	1.83	30	2.63	107	1.08	**12**	6.62
	33:03	07:06	44:03	07:01	02:02	13	4.65	10	5.46	12	4.72	17	3.78	17	4.49	16	4.07	26	2.79	**13**	6.54
	11:01	08:01	15:02	15:01	06:01	15	4.40	76	1.32	36	2.24	41	2.03	25	3.06	13	5.86	6	10.12	**14**	6.30
	01:01	06:02	37:01	10:01	05:01	8	6.60	7	7.25	5	8.53	4	10.10	12	5.62	11	6.58	21	3.56	**15**	5.27
	02:03	07:02	52:01	14:04	05:03	79	1.26	81	1.26	163	0.71	429	0.30	120	0.85	288	0.45	459	0.29	**16**	5.11
	11:01	07:02	40:01	08:03	06:01	17	4.21	30	2.24	32	2.44	33	2.30	13	4.85	12	6.14	17	4.18	**17**	5.03
	24:02	01:02	54:01	04:05	04:01	16	4.38	11	4.90	13	4.60	12	4.76	14	4.67	21	3.57	31	2.59	**18**	4.86
	33:03	14:03	44:03	13:02	06:04	7	7.39	3	11.82	6	7.38	9	5.82	6	9.52	9	7.04	40	2.29	**19**	4.45
	02:07	01:02	46:01	12:02	03:01	38	2.11	87	1.24	68	1.36	82	1.16	42	1.90	46	1.96	23	3.00	**20**	4.23

**Fig 3:**
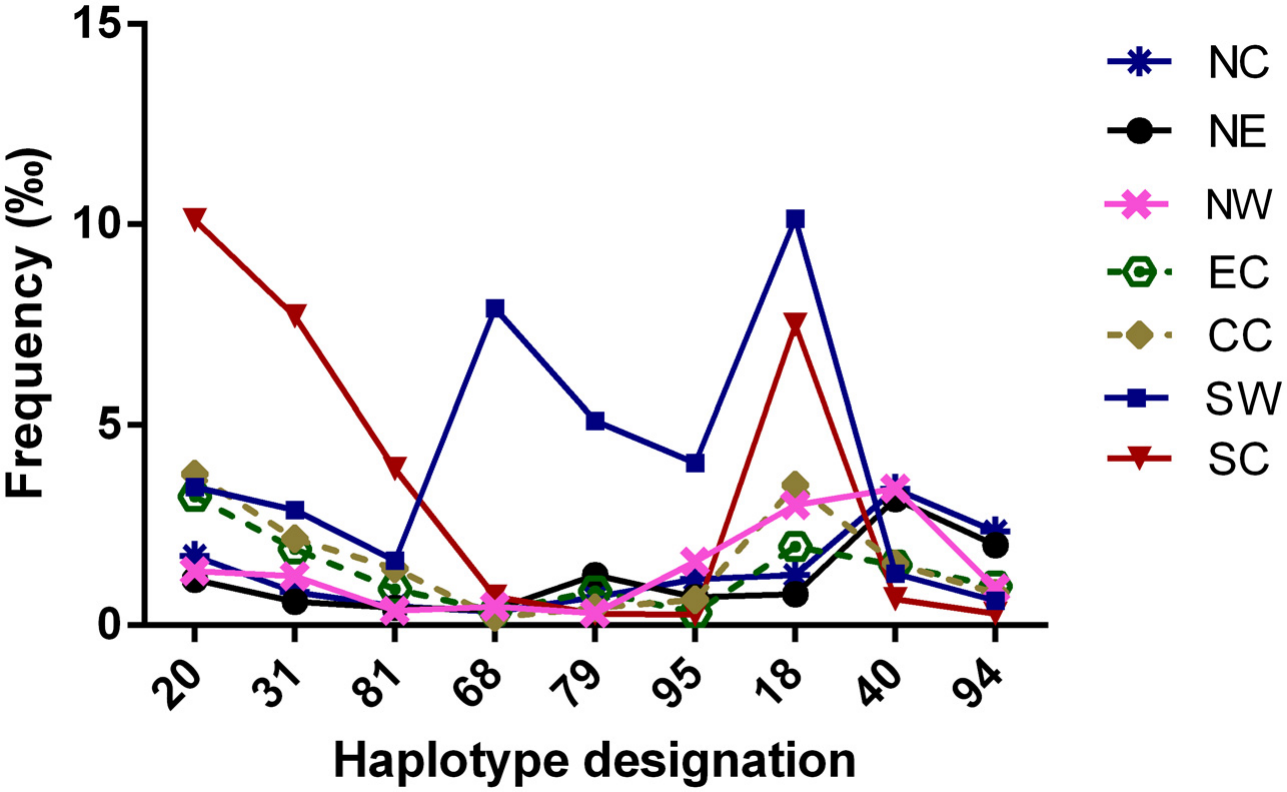
Distinctive HLA five-locus haplotypes in Southwest China and South China.

Among the 20 most common haplotypes, the 169,995 individuals exhibited quite different frequencies in the different regions. Some haplotypes were more common in the NE, NC and NW regions than in the SC and SW regions; e.g., A*30:01-C*06:02-B*13:02-DRB1*07:01-DQB1*02:02 (the 1^st^ haplotype) and A*02:01-C*03:04-B*13:01-DRB1*12:02-DQB1*03:01 (the 10^th^ haplotype). The frequencies of these haplotypes in the seven regions decreased gradually from northeast to southwest. In contrast, some other haplotypes exhibited gradually decreasing frequencies from south to north and from west to east; for example, the 4^th^ haplotype (A*11:01-C*08:01-B*15:02-DRB1*12:02-DQB1*03:01) and the 9^th^ haplotype (A*11:01-C*03:04-B*13:01-DRB1*15:01-DQB1*06:01) (Table 5, Fig 2). Additionally, A*02:07-C*01:02-B*46:01-DRB1*14:54-DQB1*05:02 (the 18^th^ haplotype) was predominant in the SC and SW regions with frequencies that were 2- to 13- fold greater than those in other five regions (Table 5). The frequencies of the 18^th^ haplotype also sequentially decreased from southwest to northeast (S11 Table). Furthermore, the 20^th^ haplotype A*02:03-C*07:02-B*38:02-DRB1*16:02- DQB1*05:02 was the most frequent (10.11%) in the SC region, where it was approximately 2.7 to 8.8 times more frequent than in all of the other six regions (Table 5, Figs 2 and 3). The 19^th^ haplotype A*11:01-C*03:04-B*13:01-DRB1*12:02-DQB1*03:01 was the sole haplotype that exhibited similar frequencies in all seven regions.

**Fig 4:**
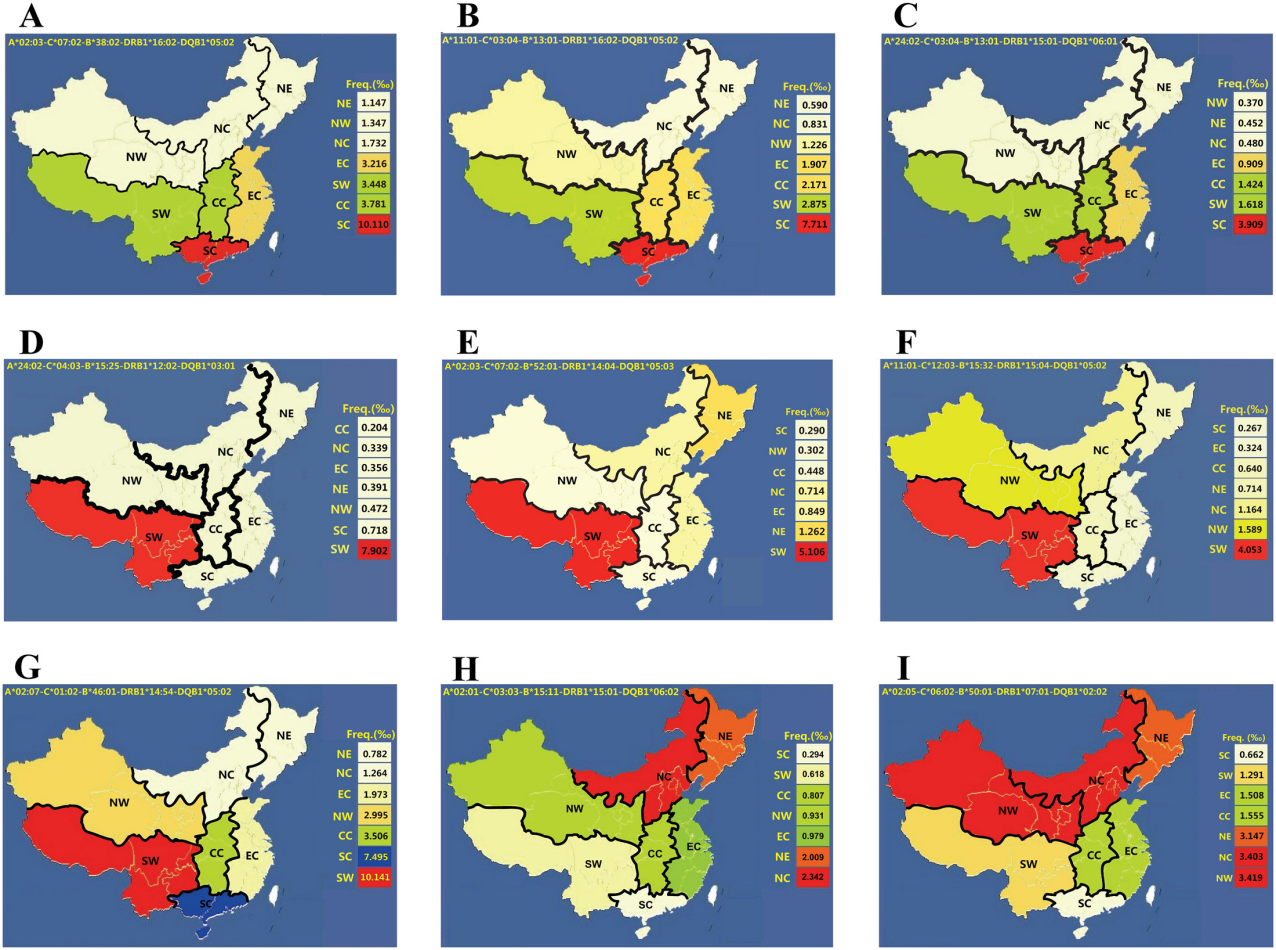
Different distributions of region-specific HLA five-locus haplotypes in the seven geographic regions of China. A: A*02:03-C*07:02-B*38:02-DRB1*16:02-DQB1*05:02 haplotype (the 20^th^ haplotype); B: A*11:01-C*03:04-B*13:01-DRB1*16:02-DQB1*05:02 haplotype (the 31^st^ haplotype); C: A*24:02-C*03:04-B*13:01-DRB1*15:01-DQB1*06:01 haplotype (the 81^st^ haplotype); D: A*24:02-C*04:03-B*15:25-DRB1*12:02-DQB1*03:01 haplotype (the 68^th^ haplotype); E: A*02:03-C*07:02-B*52:01-DRB1*14:04-DQB1*05:03 haplotype (the 79^th^ haplotype); F: A*11:01-C*12:03-B*15:32-DRB1*15:04-DQB1*05:02 haplotype (the 95^th^ haplotype); G: A*02:07-C*01:02-B*46:01-DRB1*14:54-DQB1*05:02 haplotype (the 18^th^ haplotype); H: A*02:01-C*03:03-B*15:11-DRB1*15:01-DQB1*06:02 haplotype (the 94^th^ haplotype); I: A*02:05-C*06:02-B*50:01-DRB1*07:01-DQB1*02:02 haplotype (the 40^th^ haplotype).

Among the 100 most common haplotypes in the 169,995 donors, we identified nine region-specific haplotypes in one or two to three contiguous regions. Figs 3 and 4 show the geographic distributions of nine region-specific haplotypes. First, A*02:03-C*07:02-B*38:02-DRB1*16:02-DQB1*05:02 (the 20^th^ haplotype), A*11:01-C*03:04-B*13:01-DRB1*16:02-DQB1*05:02 (the 31^st^ haplotype) and A*24:02-C*03:04-B*13:01-DRB1*15:01-DQB1*06:01 (the 81^st^ haplotype) were predominant in the SC region with frequencies of approximately 10.11‰, 7.71‰ and 3.91‰, respectively. These haplotypes were 2.7 to 13.1 times more frequent than in the other six regions (Figs 3 and 4A–4C, and S11 Table). Second, A*24:02-C*04:03-B*15:25-DRB1*12:02-DQB1*03:01 (the 68^th^ haplotype), A*02:03-C*07:02-B*52:01-DRB1*14:04-DQB1*05:03 (the 79^th^ haplotype) and A*11:01-C*12:03-B*15:32-DRB1*15:04-DQB1*05:02 (the 95^th^ haplotype) were predominant in the SW region with frequencies of approximately 7.9‰, 5.11‰, and 4.05‰, respectively (Figs 3 and 4D–4F, and S11 Table). Third, A*02:07-C*01:02-B*46:01-DRB1*14:54- DQB1*05:02 (the 18^th^ haplotype) was predominant in the SC and SW regions (Fig 4G), while A*02:01-C*03:03-B*15:11-DRB1*15:01-DQB1*06:02 (the 94^th^ haplotype) was relatively common in the NE and NC regions but rare in the SC and SW regions (Fig 4H, S11 Table). Finally, A*02:05-C*06:02-B*50:01-DRB1*07:01-DQB1*02:02 (the 40^th^ haplotype) was common in the NW, NC and NE and less common in the other four regions. The difference was highly significant (2 times to 5 times; Fig 4I, S11 Table). Additionally, no predominant haplotype was observed in either the EC or CC region.

### Verification the probabilities of HLA matching within and between geographic regions of China

In regard to HLA matching probability within and between geographic regions of China, the highest probability was observed within the SC region, almost 1.44 to 3.93 times higher than within all of the other six regions (S12 Table, Table 6). Furthermore, the matching probabilities within each region decreased from south to north. There were all high matching rates within the southern respective regions (the SC and SW regions) whereas medium or low-level matching rate within the northern respective regions (the NW, NC and NE regions). The matching rates between southern and northern regions were either medium or low. The lowest matching rate was observed between the NE region and the SW region. Additionally, high or medium-level HLA matching rates were observed between the EC region and any one of other six regions. The similar phenomenon was also discovered between the CC region and any one of other six regions (Table 6).

**Table 6:** HLA Matching within and between populations from each of the seven regions of China (matching rates).

	SW	SC	EC	CC	NE	NC	NW
**SW**	**0.0000320**	**0.0000397**	**0.0000327**	**0.0000343**	0.0000169	0.0000170	0.0000184
**SC**	**0.0000397**	**0.0000781**	**0.0000457**	**0.0000465**	0.0000182	*0*.*0000202*	*0*.*0000224*
**EC**	**0.0000327**	**0.0000457**	**0.0000542**	**0.0000483**	**0.0000352**	**0.0000308**	*0*.*0000298*
**CC**	**0.0000343**	**0.0000465**	**0.0000483**	**0.0000475**	*0*.*0000295*	*0*.*0000267*	*0*.*0000278*
**NE**	0.0000169	0.0000182	**0.0000352**	*0*.*0000295*	*0*.*0000287*	*0*.*0000243*	*0*.*0000216*
**NC**	0.0000170	*0*.*0000202*	**0.0000308**	*0*.*0000267*	*0*.*0000243*	*0*.*0000217*	0.0000197
**NW**	0.0000184	*0*.*0000224*	*0*.*0000298*	*0*.*0000278*	*0*.*0000216*	0.0000197	0.0000199

The matching rates within and between regions were plotted as a matrix. Each data point represented the matching rate between two particular regions with names in the corresponding columns and rows. The matching rates were grouped in three categories. The high-level matching rates (> 0.00003) were indicated in **bold type**. The medium-level matching rates (0.00002–0.00003) and the low-level matching rates (< 0.00002) were indicated in *italic type* and normal type respectively.

## Discussion

Our study provides high-resolution HLA allele and haplotype frequencies of the Chinese population based on a data set of 169,995 individuals. This study is the first to analyze such a large nationwide dataset that covered 31 provinces, autonomous regions, and municipalities. Compared to the previous studies [13–34], our data has significantly expanded the numbers of alleles observed at the *HLA-A*, *-B*, *-C*, *-DRB1* and -*DQB1* loci and the numbers of haplotypes in China.

We identified 156 HLA alleles as “common” alleles and 228 as “well-documented” alleles, according to the updated criteria clarified by Mack SJ [37]. There was a discrepancy between the HLA CWD alleles observed in our study and the CWD 2.0.0 alleles reported by Mack SJ. For example, A*74:02, B*15:19, C*03:56 and DQB1*05:10 are well documented in Chinese individuals but are out of the CWD 2.0.0 catalogues. Conversely, A*24:17, B*15:30, C*15:09, DRB1*01:03 and DQB1*02:03 are rare in Chinese individuals, however are common alleles within the CWD 2.0.0 catalogue [37]. These results suggest that the distributions of HLA polymorphisms are region specific and race specific. HLA data at the level of a specific population or an ethic group are important as a result of the differentiation in HLA frequencies that has occurred among human population migrations. We will discuss these differences further in other paper. Furthermore, we divided China into the seven geographic regions and were able to identify HLA alleles that were uniquely common in a single region while virtually absent from other regions. The rise and maintenance of region-specific HLA polymorphisms might be the consequences of local evolutionary selection pressure and geographic barriers. Any novel alleles might have been positively selected to enlarge the peptide-binding repertoire to counter environmental challenges. Additionally, some HLA alleles might have relatively short evolutionary histories and might be confined to regional populations, because of geographic barriers.

To analyze the regional differences in HLA genetic diversity, seven geographic regions were used for regional partitioning (Fig 1), rather than a division into southern and northern China for the following reasons. The boundary between northern China and southern China is generally believed to be the Huai River-Qin Mountains Line. However, this boundary seems to be ambiguous. First, it does not follow provincial boundaries; it cuts through Shaanxi, Henan, Anhui, and Jiangsu provinces. Second, the region around Nanyang city, Henan province, lies in the gap in which the Qin has ended and the Huai River has not yet begun. Third, central Anhui and Jiangsu lie south of the Huai River but north of the Yangtze River, which makes their classifications ambiguous as well. Moreover, the Yangtze River is also believed to be the boundary between southern and northern China by some scholars [38]. Additionally, historical population migration might also blur the boundary between northern and southern China. For example, the Song dynasty (A.D.960-1279) was established in Kaifeng, Henan province (Northern China); later, the capital city was moved to Hangzhou, Zhejiang province (Southern China) because of invasions of Jurchens and Mongols. During Song Dynasty, decades of warfare caused a massive migration from northern to southern China. Obviously, it is difficult to distinguish volunteers with southern origins from those with northern origins in the provinces of Henan, Shaanxi, Jiangsu, and Anhui. At the same time, it is extremely complicated to compare the regional differences among the 31 provinces, autonomous regions, and municipalities based on a large dataset. Our intention was to perform a preliminary analysis of the regional distributions of HLA diversity based on the traditional seven geographic regions of China. Furthermore, HLA diversity studies at the provincial or even local city level will be discussed in a follow-up paper.

Among the most common five-locus haplotypes, A*30:01-C*06:02-B*13:02-DRB1*07:01-DQB1*02:02 (the 1^st^ haplotype), A*02:07-C*01:02-B*46:01-DRB1*09:01-DQB1*03:03 (the 2^nd^ haplotype) and A*33:03-C*03:02-B*58:01-DRB1*03:01-DQB1*02:01 (the 3^rd^ haplotype) covered the most frequent five-locus haplotypes of the seven regions. Moreover, the 1^st^ haplotype was the most common in Northeast China (4.81%), and was also reported with a higher frequency of 2.68% in Korean [39], which lies adjacent to Northeast China. Korean and Northeast Chinese might have shared genetic origins. In contrast, the 2^nd^ and 3^rd^ haplotypes were predominant in Southern China (South China: 3.39%, 4.62%), which are similar to the finding of previous report [16]. Additionally, the 2^nd^ and 3^rd^ haplotypes are relatively common in Vietnam [40] with frequencies of 2.0% and 3.5%, respectively, and most frequent in Northeast Thais (3.4%, 4.6%) [41]. The 3^rd^ haplotype were also reported in Italian and Turkish living in Germany, with lower frequencies of 0.29% and 0.25% [42,43]. These observations support the hypothesis that the southern population in East Asia (including the Southern Chinese populations) and Southeast Asia might have the same ancestors [44].

As described in the results, nine of the HLA five-locus haplotypes exhibited the following clear regional characteristics (Figs 3 and 4): 1. some haplotypes displayed a decreasing pattern from the southwest to the northeast or the opposite pattern, such as the 18^th^ and 94^th^ haplotypes; 2. some haplotypes seemed to be a decreasing pattern from south to north, or exhibit the opposite direction, such as the 20^th^ and 40^th^ haplotypes; 3. some haplotypes were identified as region-specific haplotypes with frequencies that were extremely high only in a single region, such as the 20^th^, 31^st^, 81^st^, 68^th^, 79^th^, and 95^th^ haplotypes; and 4. some haplotypes exhibited wider regional characteristics and had relatively higher frequencies in several adjacent regions, such as the 18^th^, 94^th^, and 40^th^ haplotypes.

Among these distinctive haplotypes, A*02:03-C*07:02-B*38:02-DRB1*16:02-DQB1*05:02 (the 20^th^ haplotype), which was identified as one of South China- specific haplotype (1.01%) in the present study, has also been reported in southern Chinese Han population (0.54%) [16] and in mixed of northern and southern Chinese Han individuals (0.55%) [45]. These findings suggest that the 20^th^ haplotype is prominent in southern China. Moreover, A*24:02-C*04:03-B*15:25-DRB1*12:02-DQB1*03:01 (the 68^th^ haplotype), one of Southwest China-specific haplotypes identified in this study, has also been reported in Vietnam [40], with a higher frequency of 1.1%. Obviously, the frequency of this haplotype in Vietnam is similar to that in Southwest China (0.79%), supporting the hypothesis that populations from the south China and Vietnamese might derive from the similar origins. The 68^th^ haplotype might be a significant haplotype unique for some Southeast-Asia and East-Asia regions including Vietnam, and Southwest China. Additionally, the 20^th^ haplotype and A*02:03-C*07:02-B*52:01-DRB1*14:04-DQB1*05:03 (the 79^th^ haplotype), another Southwest China-specific haplotype identified in our study, have also been observed in Asians living in the United State with frequencies of 0.325%, 0.108%, respectively [46], similar to those in the EC region observed in the present study, while A*11:01-C*03:04-B*13:01-DRB1*16:02-DQB1*05:02 (the 31^st^ haplotype), another South China-specific haplotypes observed in our study, has also been discovered in Asians and Hispanics living in America, with frequencies of 0.108%, 0.156%, respectively [46], similar to the frequency in the Northwest China (0.12%) observed in our study (S11 Table). The Asian populations in the NMDP registry are very heterogeneous populations, while our population in each of the regions is a quite homogeneous.

A*02:07-C*01:02-B*46:01-DRB1*14:54-DQB1*05:02 (the 18th haplotype) was identified as one region-specific haplotype of SW and SC regions, with frequencies of 1.01% and 0.75%, respectively. The 18^th^ haplotype has not been previously reported, which is likely due to the fact that the earlier HLA typing did not distinguish DRB1*14:01 from DRB1*14:54 in most laboratories. As a result, DRB1*14:54 is not reported in the majority of references [14–17,19,39–41,45]. DRB1*14:01:01 differed from DRB1*14:54 in one nucleotide of the exon 3 region, which results in an amino acid change [47]. Ambiguous results are caused by the genotyping of only the second exon of HLA-DRB1 locus. To distinguish DRB1*14:54 from DRB1*14:01 and to investigate the distribution of DRB1*14:54, we sequenced exon 3 of the DRB1 locus in the individuals involved. The frequency of DRB1*14:54 (3.20%) was far higher than that of DRB1*14:01 (0.005%) in our studied population. Additionally DRB1*14:54 was relatively predominant in the Southwest (5.26%) and South China regions (4.56%), which is similar to previous results [23,24,48,49]. While A*02:07-C*01:02-B*46:01-DRB1*14:01-DQB1*05:02 haplotype was found as expected in the southern Chinese Han population with a higher frequency of 1.08% as published by Gao SQ [16], these authors did not identify the DRB1*14:54. The frequency distribution of this haplotype is similar to that of the 18^th^ haplotype in the South (0.75%) and Southwest China (1.01%) regions in the present study. To the best of our knowledge, the 40^th^, 81^st^, 94^th^, and 95^th^ haplotypes, other four region-specific haplotypes identified in the present study, have not been previously reported.

Furthermore, some alleles were found in multiple regions that had different distinctive haplotypic associations, which suggest that these alleles might be ancient and might have diverged through recombination at different times in different regions to generate new haplotypes. For example, A*02:03 was observed in both the 20^th^ haplotype (the distinctive haplotype of the SC region) and the 79^th^ haplotype (the distinctive haplotype of the SW region). A*02:03 exhibited the higher frequencies in South china (7.7%) and Southwest China (5.5%). Besides, its frequencies decreased gradually from south to north. This allele was most frequent in Guangxi province (11.2%), followed by Guangdong province (7.4%), and Yunnan province (6.2%) in the present study (data not shown); these percentages are similar to those reported in the literature [15,16,26,27,29,30,32–34]. A*02:03 has also been observed to have lower frequencies in German (0.011%) [10], Japan (0.1%) [50] and South Korea (0.55%) [39,51], and to have medium frequencies in Malaysia (2%-4%) [52] and Indonesia Java (3.6%) [53]. Whereas A*02:03 were predominant in the Northeast Thailand [41] and Vietnam[40], with higher frequencies of 10.6%, 7.9%, respectively, which are in close proximity to Southwest China and South China. It has been suggested that A*02:03 expanded dramatically in the southeast region of Asia including Thailand, Vietnam and the south parts of China after early humans settled in Southeast Asia. Additionally, A*02:03 is known to be serologically different from other common A*02 alleles such as A*02:01. It is reasonable to hypothesize that the rise of A*02:03 in that region might have played a critical role in adapting to the new environment by increasing the peptide binding reservoir or by other means. As a consequence of the A*02:03 expansion, new haplotypes associated with A*02:03 were established.

Our results revealed that several characteristic haplotypes were primarily located in southern China (the Southwest China and South China regions) and northern China (the Northwest, Northeast, and North China regions). Meanwhile, we also found that the HLA matching rates were higher within the southern respective regions (the SC and SW regions) than within the northern respective regions (the NW, NC and NE regions). Additionally, the low-level HLA matching rates were primarily observed between the northern regions and the southern regions. These findings support the hypothesis that the Chinese Nation comprises two distinct populations of the north and south. This hypothesis agrees well with previous studies [38,44,54]. In contrast, no region-specific haplotypes were observed in the Central China or East China regions. Moreover, high HLA matching rates were observed not only between the EC region and the southern respective regions (the SC and SW regions) but also between the EC region and the northern respective regions (the NC and NE regions). The similar phenomenon was also discovered in the CC region. These regions (the EC and CC regions) cover provinces that are crossed by the traditional border between southern and northern China; i.e., the Huai River-Qin Mountain Line. It has been suggested that residents of these regions might possess characteristics of both North and South Chinese. That is to say, HLA diversities of the EC and CC region’s population may cover those of majorities of northern and southern Chinese. Consequently, it may be worth to consider expanding marrow donor pool in these regions to further improve coverage of HLA diversity throughout China.

Our results regarding the HLA matching rates also showed that the highest probability was observed within the SC region and the matching probabilities within each region decreased from south to north. It suggests that SC region population are less HLA polymorphic than other regions’ population. Additionally, the low-level matching rates were primarily observed between the northern regions and the southern regions (the SW region). These differences in HLA matching rates between regions may be due to differences in nationality composition, ancestry and shift and migration of the population of each geographic region. Based on the HLA matching rates within each region or between different regions (Table 6) we can estimate the relative likelihood to find a perfect donor for a particular patient from a given region. Further research is needed to explore how the differences in HLA matching rates impact on the search of potential bone marrow donors within each Chinese region.

In the present study, the proportions of Han Chinese in the seven regions were 84.5% to 99.5%. Obviously, the Han Chinese population is a predominant population in all studied regions, while the individuals of minority nationalities account for less than 6 percent of the observed individuals. Additionally, the southern minorities are more closely related to the southern Han Chinese, while the northern minorities and northern Han Chinese group together, according to the clustering analysis published by Chen [54]. Accordingly, minority nationalities might have no influence on the major conclusion of the present studies.

The HLA system is known to have been under strong selection for thousands of years due to its important role in the immune response, and the HLA haplotype is a useful genetic marker for the identification of closely related population groups, because each characteristic haplotype is considered to have a single origin [55]. Our results might be useful for studies of origins of nationalities, anthropogenesis, and population genetics.

In conclusion, we preliminarily described and analyzed characteristics and regional differences of HLA diversity distributions based on 169,995 Chinese individuals who were typed at high resolution for the *HLA-A*, *HLA-B*, *HLA-C*, and *HLA-DRB1* and *HLA-DQB1* genes. Although the individuals from each of the geographic regions shared certain common alleles and haplotypes, the populations residing in Southwest and South China maintained their distinctive genetic characteristics. These findings suggest that selective recruitment of stem cell donors with characteristic human leukocyte alleles and haplotypes is necessary. Furthermore, the HLA diversity analysis of seven geographic regions may provide meaningful tools to optimize the new marrow donor recruitment strategy and donor search strategy.

## Materials and Methods

### Ethics statement

The study was approved by the review board of the China Marrow Donor Program, Beijing, China. All patients provided written informed consent.

### Sample population

Our analysis consisted of 169,995 healthy individuals from CMDP volunteers, who were recruited from January 2010 to January 2012. All volunteers were recruited at local CMDP recruitment centers throughout 31 provinces, autonomous regions, and municipalities (i.e., Heilongjiang, Jilin, Liaoning, Beijing, Tianjin, Hebei, Shanxi, Inner Mongolia, Xinjiang, Qinghai, Gansu, Ningxia, Shaanxi, Shanghai, Shandong, Jiangsu, Anhui, Zhejiang, Jiangxi, Fujian, Henan, Hubei, Hunan, Guangdong, Guangxi, Hainan, Chongqing, Tibet, Yunnan, Sichuan, and Guizhou). Upon recruitment, volunteers were asked to fulfill personal information including their birthplace. In our study, the individuals were classified according to where they were born. During the recruitment, the individuals who recruited from a province include both the native-born (85%) and immigrants (15%). All volunteers were 18 to 50 years old, and males (47.2%) and females (52.8%) were included. Among the 169,995 volunteers, 94.6% were Han Chinese and 5.4% were minority Chinese.

### HLA genotyping

All of the individuals were typed at high-resolution levels for *HLA-A*, *-B*, *-C*, *-DRB1* and-*DQB1*. High-resolution HLA typings were performed using two methods: next-generation high-throughput sequence based typing (NGS, n = 117,993), and Sanger sequencing-based typing (SBT, n = 52,002). HLA typing was performed by the CMDP-accredited laboratories. The alleles were generally given in a two-field form, and ambiguities within the relevant exons were resolved with group-specific sequencing primers (GSSP) or high resolution sequence specific primers (SSP). Additionally, alleles with synonymous mutations inside or outside the relevant exons were merged. Examples are as follows: A*01:01:01:01 and A*01:01:02 (not identical within exons 2 and 3) were merged to A*01:01, and A*32:01:01 and A*32:01:02 (identical within exons 2 and 3) were merged to A*32:01. However, alleles that differed in nonsynonymous mutations outside the relevant exons were not yet merged. For example, A*02:03 and A*02:253 (identical within exons 2 and 3) had to be identified by sequencing exon 4.

Based on SBT method, exons 2, 3 and 4 were sequenced for HLA-A, -B, and HLA-C, exon 2 was sequenced for HLA-DRB1, and exons 2 and 3 were sequenced for HLA-DQB1. Based on NGS based typing method, exons 1 to 7 were sequenced for HLA-A, -B and HLA-C, exon 2 was sequenced for HLA-DRB1, and exons 2 and 3 were sequenced for HLA-DQB1. Briefly, next-generation clonal sequencing of the exonic amplicons was performed using a reliable, cost-effective and high-throughput sequence-based typing (RCHSBT) method [11]. High-resolution sequencing to obtain HLA genotypes was performed as described in detail earlier [11].

### Criteria for CWD alleles

In this study, the criteria of Mack SJ et al. were adopted [37]. Common alleles were those that have been observed at frequencies >0.001 in reference populations of at least 1500 individuals [37,56]. Alleles detected five times via SBT in unrelated individuals or those that were detected three times via SBT in unrelated individuals sharing a haplotype were assigned to the well-documented category [37]. The remaining alleles were considered rare alleles [56].

### Geographic partitioning

Traditionally from the north to the south, China can be divided into seven geographical regions (Fig 1). These partitions are as follows: Northeast China (NE) includes Heilongjiang, Jilin, and Liaoning; North China (NC) consists of Beijing, Tianjin, Hebei, Shanxi, and Inner Mongolia; Northwest China (NW) is comprised of Xinjiang, Qinghai, Gansu, Ningxia, and Shaanxi; East China (EC) comprises Shanghai, Shandong, Jiangsu, Anhui, Zhejiang, Jiangxi, and Fujian; Central China (CC) has three provinces, including Henan, Hubei, Hunan; South China (SC) covers Guangdong, Guangxi, Hainan; and Southwest China (SW) is made up of Chongqing, Tibet, Yunnan, Sichuan, Guizhou. Additionally, Taiwan, Hong Kong and Macau were not included in this study.

To identify the characteristic distributions of the HLA diversities, regional partitioning was performed based on the seven geographical regions. We used the geographical regions partitioning to group 169,995 unrelated volunteers from 31 provinces, autonomous regions and municipalities into seven groups, i.e., Northeast China (NE, n = 12,493), North China (NC, n = 27,819), Northwest China (NW, n = 14,409), East China (EC, n = 51,132), Central China (CC, n = 24,432), South China (SC, n = 18,657) and Southwest China (SW, n = 21,053) (S10 Table). Furthermore, the proportions of Han Chinese people in each region were as follows: 99.5% (EC), 96.5% (CC), 95.4% (NE), 94.2% (NW), 92.9% (NC), 92.3% (SC), and 84.5% (SW).

According to the 2010 National Population Census [35], the population of China was approximately 1.34 billion (not including the population in the Hong Kong Special Administrative Region, the Macao Special Administrative Region and the Taiwan province). The total populations of each of the seven geographic regions were as follows: approximately 109.5 million people in Northeast China, 96.6 million people in Northwest China, 164.8 million people in North China, 392.9 million people in East China, 216.9 million people in Central China, 159.0 million people in South China and 192.9 million people in Southwest China. Consequently, the research samples of each region observed in this study accounted for approximately 0.11 ‰ to 0.17 ‰ of the total populations of the respective regions (S10 Table).

### Statistical analysis

The allele frequencies were calculated by direct counting, and Hardy-Weinberg equilibrium tests (heterozygosity and p value) were performed for each of the five HLA loci using the Arlequin software package [57]. The input parameters for the Markov Chain Monte Carlo test described by Guo and Thompson [58] were 10^6^ Markov chain steps and 2*10^3^ dememorization steps. Values of p <0.05 were regarded as significant.

The haplotype frequencies were estimated with the expected maximum likelihood estimation algorithm with the Arlequin software [57]. Furthermore, linkage disequilibrium was also calculated for two-locus haplotypes.

The pairwise comparisons were carried out using Cervus 3.0.7 software [59,60], and a 10/10 alleles at high resolution level for HLA-A, -B, -C, -DRB1 and -DQB1 was used as matching criteria. The matching rate was calculated in Winpepi 11.44 [60,61], and the odds ratio and chi-square were calculated using SPSS 16.0.

## Supporting Information

S1 Table HLA-A allele frequencies among the 169,995 CMDP registry donors. (DOCX)

S2 Table HLA-B allele frequencies among the 169,995 CMDP registry donors. (DOCX)

S3 Table HLA-C allele frequencies among the 169,995 CMDP registry donors. (DOCX)

S4 Table HLA-DRB1 allele frequencies among the 169,995 CMDP registry donors. (DOCX)

S5 Table HLA-DQB1 allele frequencies among the 169,995 CMDP registry donors. (DOCX)

S6 Table Common (F-est.>1‰) HLA two-locus haplotypes among the 169,995 CMDP registry donors. (DOCX)

S7 Table Common (freq.>1‰) HLA three-locus haplotypes among the 169,995 CMDP registry donors. (DOCX)

S8 Table Common (freq.>1‰) HLA four-locus haplotypes among the 169,995 CMDP registry donors. (DOCX)

S9 Table Common (freq.>1‰) HLA five-locus haplotypes among the 169,995 CMDP registry donors. (DOCX)

S10 Table The numbers of donors from each of the geographic regions of China. (DOCX)

S11 Table Frequencies of several distinctive haplotypes in each of the geographic regions. (DOCX)

S12 Table HLA matches within each region of China. (DOCX)
