# Supplementary material for: High-Resolution Analyses of Human Leukocyte Antigens Allele and Haplotype Frequencies Based on 169,995 Volunteers from the China Bone Marrow Donor Registry Program
Source: PLoS One. 2015 Sep 30;10(9):e0139485. doi: 10.1371/journal.pone.0139485 (PMC4589403; doi:10.1371/journal.pone.0139485)
Supplement: S1 Table — (DOCX) [file pone.0139485.s001.docx]

**Supporting information**

**S1Table.**  HLA-A allele frequencies among the 169,995 CMDP registry donors

| Allele | Freq (%) | Allele | Freq (%) | Allele | Freq (%) | Allele | Freq (%) | Allele | Freq (%) |
| --- | --- | --- | --- | --- | --- | --- | --- | --- | --- |
| A*01:01 | 3.4036 | A*02:279 | 0.0003 | A*11:56 | 0.0006 | A*24:21 | 0.0006 | A*31:06 | 0.0006 |
| A*01:03 | 0.0144 | A*02:28 | 0.0009 | A*11:60 | 0.0015 | A*24:28 | 0.0003 | A*31:13 | 0.0024 |
| A*01:28 | 0.0003 | A*02:293Q | 0.0003 | A*11:61 | 0.0006 | A*24:30 | 0.0059 | A*31:17 | 0.0009 |
| A*01:43 | 0.0003 | A*02:295 | 0.0003 | A*11:69N | 0.0006 | A*24:33 | 0.0006 | A*31:31 | 0.0003 |
| A*01:78 | 0.0021 | A*02:311 | 0.0003 | A*11:74 | 0.0003 | A*24:52 | 0.0012 | A*31:33 | 0.0006 |
| A*02:01 | 12.2898 | A*02:42 | 0.0012 | A*11:75 | 0.0006 | A*24:53 | 0.0006 | A*31:46 | 0.0003 |
| A*02:02 | 0.0050 | A*02:48 | 0.0071 | A*11:76 | 0.0003 | A*24:59 | 0.0015 | A*32:01 | 1.2792 |
| A*02:03 | 3.4963 | A*02:53N | 0.0518 | A*11:77 | 0.0003 | A*24:62 | 0.0003 | A*33:01 | 0.1553 |
| A*02:05 | 0.3347 | A*02:59 | 0.0006 | A*11:79 | 0.0003 | A*24:63 | 0.0003 | A*33:03 | 7.9311 |
| A*02:06 | 5.1996 | A*02:64 | 0.0003 | A*11:80 | 0.0003 | A*24:64 | 0.0029 | A*33:06 | 0.0003 |
| A*02:07 | 8.5035 | A*02:79 | 0.0012 | A*11:87 | 0.0006 | A*24:68 | 0.0077 | A*33:08 | 0.0035 |
| A*02:08 | 0.0003 | A*02:80 | 0.0012 | A*11:88 | 0.0059 | A*24:82 | 0.0003 | A*33:11 | 0.0003 |
| A*02:09 | 0.0382 | A*02:90 | 0.0035 | A*23:01 | 0.2694 | A*24:85 | 0.0018 | A*33:25 | 0.0003 |
| A*02:10 | 0.4153 | A*02:92 | 0.0003 | A*23:17 | 0.0003 | A*24:91 | 0.0003 | A*33:30 | 0.0003 |
| A*02:108 | 0.0029 | A*02:93 | 0.0077 | A*23:20 | 0.0006 | A*24:93 | 0.0009 | A*33:31 | 0.0003 |
| A*02:11 | 0.0135 | A*02:95 | 0.0006 | A*23:26 | 0.0009 | A*24:98 | 0.0006 | A*33:43 | 0.0003 |
| A*02:116 | 0.0003 | A*02:99 | 0.0018 | A*24:02 | 15.5687 | A*25:01 | 0.0221 | A*33:44 | 0.0003 |
| A*02:12 | 0.0018 | A*03:01 | 3.0013 | A*24:03 | 0.1744 | A*26:01 | 2.7969 | A*34:01 | 0.0403 |
| A*02:121 | 0.0003 | A*03:02 | 0.1985 | A*24:04 | 0.0382 | A*26:02 | 0.0156 | A*34:02 | 0.0021 |
| A*02:127 | 0.0003 | A*03:05 | 0.0003 | A*24:05 | 0.0006 | A*26:03 | 0.0215 | A*36:02 | 0.0012 |
| A*02:133 | 0.0003 | A*03:12 | 0.0003 | A*24:06 | 0.0003 | A*26:08 | 0.0006 | A*66:01 | 0.0506 |
| A*02:158 | 0.0003 | A*03:120 | 0.0003 | A*24:07 | 0.2156 | A*26:14 | 0.0006 | A*68:01 | 0.7427 |
| A*02:166 | 0.0003 | A*03:77 | 0.0032 | A*24:08 | 0.0753 | A*26:17 | 0.0006 | A*68:02 | 0.0500 |
| A*02:17 | 0.0024 | A*03:78 | 0.0003 | A*24:10 | 0.0709 | A*26:18 | 0.0088 | A*68:24 | 0.0062 |
| A*02:187 | 0.0003 | A*11:01 | 21.1430 | A*24:111 | 0.0003 | A*26:20 | 0.0091 | A*68:38 | 0.0027 |
| A*02:189 | 0.0015 | A*11:02 | 1.9168 | A*24:128 | 0.0029 | A*26:35 | 0.0006 | A*69:01 | 0.0979 |
| A*02:20 | 0.0035 | A*11:03 | 0.1185 | A*24:13 | 0.0003 | A*26:36 | 0.0012 | A*74:01 | 0.0024 |
| A*02:217 | 0.0003 | A*11:04 | 0.0050 | A*24:131 | 0.0003 | A*26:46 | 0.0003 | A*74:02 | 0.0527 |
| A*02:227N | 0.0003 | A*11:06 | 0.0009 | A*24:132N | 0.0015 | A*26:50 | 0.0006 | A*74:03 | 0.0044 |
| A*02:230 | 0.0009 | A*11:112 | 0.0003 | A*24:136 | 0.0003 | A*29:01 | 0.8424 | A*74:05 | 0.0024 |
| A*02:24 | 0.0003 | A*11:12 | 0.0003 | A*24:141 | 0.0003 | A*29:02 | 0.0665 | A*74:13 | 0.0009 |
| A*02:245 | 0.0003 | A*11:14 | 0.0006 | A*24:15 | 0.0003 | A*29:10 | 0.0024 |  |  |
| A*02:249 | 0.0009 | A*11:19 | 0.0003 | A*24:150 | 0.0006 | A*30:01 | 5.4340 |  |  |
| A*02:251 | 0.0006 | A*11:20 | 0.0003 | A*24:151 | 0.0003 | A*30:02 | 0.0277 |  |  |
| A*02:253 | 0.0003 | A*11:27 | 0.0003 | A*24:152 | 0.0009 | A*30:04 | 0.0659 |  |  |
| A*02:256 | 0.0009 | A*11:32 | 0.0012 | A*24:154 | 0.0003 | A*30:18 | 0.0218 |  |  |
| A*02:259 | 0.0003 | A*11:36 | 0.0024 | A*24:161 | 0.0003 | A*30:35 | 0.0003 |  |  |
| A*02:264 | 0.0029 | A*11:41 | 0.0003 | A*24:162 | 0.0003 | A*30:38 | 0.0003 |  |  |
| A*02:269 | 0.0003 | A*11:48 | 0.0003 | A*24:17 | 0.0009 | A*30:43 | 0.0003 |  |  |
| A*02:277 | 0.0003 | A*11:55 | 0.0006 | A*24:20 | 0.2532 | A*31:01 | 3.2986 |  |  |

Note: The allele frequencies of 0.0003% indicate alleles that were observed only once across all donors
