# Supplementary material for: High-Resolution Analyses of Human Leukocyte Antigens Allele and Haplotype Frequencies Based on 169,995 Volunteers from the China Bone Marrow Donor Registry Program
Source: PLoS One. 2015 Sep 30;10(9):e0139485. doi: 10.1371/journal.pone.0139485 (PMC4589403; doi:10.1371/journal.pone.0139485)
Supplement: S2 Table — (DOCX) [file pone.0139485.s002.docx]

**Supporting information**

**S2 Table.**  HLA-B allele frequencies among the 169,995 CMDP registry donors

| Allele | Freq (%) | Allele | Freq (%) | Allele | Freq (%) | Allele | Freq (%) | Allele | Freq (%) |
| --- | --- | --- | --- | --- | --- | --- | --- | --- | --- |
| B*07:02 | 2.1842 | B*15:25 | 0.6244 | B*35:29 | 0.0003 | B*40:69 | 0.0006 | B*51:36 | 0.0129 |
| B*07:05 | 0.7321 | B*15:27 | 0.8094 | B*35:30 | 0.0015 | B*40:70 | 0.0003 | B*51:39 | 0.0018 |
| B*07:06 | 0.0577 | B*15:29 | 0.0094 | B*35:42 | 0.0021 | B*40:72 | 0.0003 | B*51:51 | 0.0003 |
| B*07:10 | 0.0044 | B*15:30 | 0.0006 | B*35:54 | 0.0003 | B*40:75 | 0.0006 | B*51:78 | 0.0003 |
| B*07:31 | 0.0003 | B*15:32 | 0.2241 | B*35:64 | 0.0003 | B*40:78 | 0.0132 | B*52:01 | 2.8542 |
| B*07:48 | 0.0009 | B*15:34 | 0.0003 | B*35:85 | 0.0003 | B*40:81 | 0.0006 | B*52:04 | 0.0003 |
| B*07:81 | 0.0003 | B*15:35 | 0.0200 | B*37:01 | 1.3468 | B*40:84 | 0.0024 | B*52:06 | 0.0003 |
| B*07:91 | 0.0003 | B*15:38 | 0.0071 | B*37:02 | 0.0003 | B*40:87 | 0.0003 | B*52:11 | 0.0009 |
| B*08:01 | 0.8621 | B*15:39 | 0.0006 | B*37:04 | 0.0035 | B*40:97 | 0.0079 | B*53:01 | 0.0329 |
| B*08:09 | 0.0006 | B*15:46 | 0.0462 | B*38:01 | 0.4888 | B*41:01 | 0.1018 | B*54:01 | 3.0389 |
| B*13:01 | 5.0131 | B*15:56 | 0.0003 | B*38:02 | 2.6130 | B*41:02 | 0.0200 | B*54:16 | 0.0003 |
| B*13:02 | 5.7690 | B*15:58 | 0.0935 | B*38:15 | 0.0035 | B*42:01 | 0.0109 | B*54:17 | 0.0071 |
| B*13:15 | 0.0003 | B*15:65 | 0.0009 | B*39:01 | 1.7842 | B*42:02 | 0.0050 | B*55:01 | 0.0894 |
| B*13:16 | 0.0021 | B*15:66 | 0.0003 | B*39:04 | 0.0003 | B*44:02 | 0.9774 | B*55:02 | 2.4515 |
| B*13:28 | 0.0003 | B*15:68 | 0.0047 | B*39:05 | 0.1782 | B*44:03 | 2.6295 | B*55:04 | 0.0524 |
| B*13:38 | 0.0006 | B*15:70 | 0.0003 | B*39:06 | 0.0065 | B*44:05 | 0.0062 | B*55:07 | 0.0277 |
| B*13:40 | 0.0003 | B*15:78 | 0.0009 | B*39:09 | 0.0294 | B*44:06 | 0.0003 | B*55:12 | 0.0362 |
| B*13:43 | 0.0038 | B*15:86 | 0.0009 | B*39:10 | 0.0003 | B*44:27 | 0.0021 | B*55:16 | 0.0006 |
| B*14:01 | 0.0441 | B*15:88 | 0.0006 | B*39:15 | 0.0153 | B*44:28 | 0.0003 | B*55:21 | 0.0018 |
| B*14:02 | 0.2591 | B*15:93 | 0.0003 | B*39:24 | 0.0074 | B*44:29 | 0.0003 | B*55:26 | 0.0003 |
| B*15:01 | 4.9166 | B*18:01 | 0.3991 | B*39:31 | 0.0062 | B*44:47 | 0.0003 | B*55:30 | 0.0009 |
| B*15:02 | 3.5445 | B*18:02 | 0.0562 | B*39:36 | 0.0012 | B*44:87 | 0.0003 | B*55:42 | 0.0003 |
| B*15:03 | 0.0391 | B*18:03 | 0.0024 | B*39:38Q | 0.0006 | B*45:01 | 0.1124 | B*55:43 | 0.0003 |
| B*15:05 | 0.1650 | B*27:02 | 0.0194 | B*39:58 | 0.0006 | B*46:01 | 10.2206 | B*55:48 | 0.0006 |
| B*15:07 | 0.3180 | B*27:03 | 0.0091 | B*40:01 | 9.9559 | B*46:08 | 0.0003 | B*55:50 | 0.0003 |
| B*15:08 | 0.0212 | B*27:04 | 0.9442 | B*40:02 | 1.9298 | B*46:09 | 0.0006 | B*56:01 | 0.5180 |
| B*15:09 | 0.0018 | B*27:05 | 0.7656 | B*40:03 | 0.2212 | B*46:14 | 0.0003 | B*56:03 | 0.0474 |
| B*15:10 | 0.0077 | B*27:06 | 0.0550 | B*40:06 | 3.2322 | B*46:19 | 0.0003 | B*56:04 | 0.0838 |
| B*15:105 | 0.0003 | B*27:07 | 0.1400 | B*40:11 | 0.0021 | B*46:27 | 0.0006 | B*56:09 | 0.0006 |
| B*15:11 | 1.8315 | B*27:14 | 0.0015 | B*40:114 | 0.0003 | B*47:01 | 0.0206 | B*56:10 | 0.0077 |
| B*15:12 | 0.2665 | B*27:15 | 0.0053 | B*40:122 | 0.0006 | B*48:01 | 2.4615 | B*56:11 | 0.0003 |
| B*15:128 | 0.0003 | B*27:24 | 0.0212 | B*40:125 | 0.0006 | B*48:03 | 0.1603 | B*56:14 | 0.0012 |
| B*15:13 | 0.0532 | B*27:25 | 0.0074 | B*40:141 | 0.0009 | B*48:04 | 0.0035 | B*56:18 | 0.0003 |
| B*15:134 | 0.0003 | B*27:27 | 0.0003 | B*40:147 | 0.0003 | B*48:06 | 0.0009 | B*56:21 | 0.0006 |
| B*15:15 | 0.0003 | B*27:36 | 0.0035 | B*40:150 | 0.0068 | B*48:16 | 0.0006 | B*56:27 | 0.0006 |
| B*15:152 | 0.0003 | B*27:40 | 0.0003 | B*40:151 | 0.0006 | B*49:01 | 0.1530 | B*57:01 | 1.0921 |
| B*15:17 | 0.1885 | B*27:44 | 0.0003 | B*40:152 | 0.0003 | B*49:11 | 0.0003 | B*57:02 | 0.0024 |
| B*15:178 | 0.0041 | B*27:69 | 0.0029 | B*40:153 | 0.0003 | B*50:01 | 0.6200 | B*57:03 | 0.0050 |
| B*15:18 | 1.3453 | B*35:01 | 2.8524 | B*40:25 | 0.0003 | B*51:01 | 5.6363 | B*57:29 | 0.0018 |
| B*15:19 | 0.0368 | B*35:02 | 0.1971 | B*40:33 | 0.0003 | B*51:02 | 1.0565 | B*58:01 | 5.8693 |
| B*15:192 | 0.0009 | B*35:03 | 1.1059 | B*40:39 | 0.0003 | B*51:05 | 0.0003 | B*58:11 | 0.0003 |
| B*15:198 | 0.0068 | B*35:04 | 0.0009 | B*40:40 | 0.0703 | B*51:06 | 0.0097 | B*58:12 | 0.0003 |
| B*15:201 | 0.0003 | B*35:05 | 0.2644 | B*40:43 | 0.0009 | B*51:07 | 0.0329 | B*58:19 | 0.0003 |
| B*15:209N | 0.0003 | B*35:08 | 0.1394 | B*40:48 | 0.0009 | B*51:08 | 0.0371 | B*59:01 | 0.0853 |
| B*15:21 | 0.0288 | B*35:10 | 0.0003 | B*40:49 | 0.0006 | B*51:09 | 0.0035 | B*67:01 | 0.7468 |
| B*15:212 | 0.0003 | B*35:107 | 0.0003 | B*40:50 | 0.0032 | B*51:104 | 0.0003 | B*67:03 | 0.0009 |
| B*15:214 | 0.0003 | B*35:11 | 0.0024 | B*40:54 | 0.0003 | B*51:21 | 0.0024 | B*73:01 | 0.0109 |
| B*15:215 | 0.0003 | B*35:137 | 0.0003 | B*40:55 | 0.0135 | B*51:22 | 0.0009 | B*81:02 | 0.1018 |
| B*15:220 | 0.0038 | B*35:147 | 0.0003 | B*40:60 | 0.0003 | B*51:34 | 0.0006 |  |  |
