# Supplementary material for: High-Resolution Analyses of Human Leukocyte Antigens Allele and Haplotype Frequencies Based on 169,995 Volunteers from the China Bone Marrow Donor Registry Program
Source: PLoS One. 2015 Sep 30;10(9):e0139485. doi: 10.1371/journal.pone.0139485 (PMC4589403; doi:10.1371/journal.pone.0139485)
Supplement: S3 Table — (DOCX) [file pone.0139485.s003.docx]

**Supporting information**

**S3 Table.**  HLA-C allele frequencies among the 169,995 CMDP registry donors

| Allele | Freq (%) | Allele | Freq (%) | Allele | Freq (%) | Allele | Freq (%) | Allele | Freq (%) |
| --- | --- | --- | --- | --- | --- | --- | --- | --- | --- |
| C*01:02 | 15.5584 | C*03:41 | 0.0006 | C*06:04 | 0.0009 | C*07:49 | 0.0003 | C*14:20 | 0.0009 |
| C*01:03 | 0.6197 | C*03:43 | 0.0009 | C*06:14 | 0.0003 | C*07:51 | 0.0012 | C*14:23 | 0.0006 |
| C*01:06 | 0.0824 | C*03:45 | 0.0003 | C*06:17 | 0.0003 | C*07:56 | 0.0012 | C*14:24 | 0.0003 |
| C*01:08 | 0.0200 | C*03:48 | 0.0012 | C*06:23 | 0.0003 | C*07:63 | 0.0100 | C*14:25 | 0.0018 |
| C*01:09 | 0.0003 | C*03:49 | 0.0003 | C*06:24 | 0.0003 | C*07:66 | 0.0844 | C*15:02 | 3.3798 |
| C*01:10 | 0.0012 | C*03:56 | 0.0150 | C*06:30 | 0.0003 | C*07:67 | 0.0053 | C*15:03 | 0.0003 |
| C*01:17 | 0.0021 | C*03:58 | 0.0003 | C*06:35 | 0.0003 | C*07:76 | 0.0003 | C*15:04 | 0.0350 |
| C*01:22 | 0.0018 | C*03:64 | 0.0009 | C*06:53 | 0.0003 | C*08:01 | 8.4623 | C*15:05 | 0.7427 |
| C*01:30 | 0.0006 | C*03:69 | 0.0003 | C*07:01 | 0.5559 | C*08:02 | 0.3035 | C*15:06 | 0.0021 |
| C*01:40 | 0.0006 | C*03:76 | 0.0009 | C*07:02 | 15.1519 | C*08:03 | 0.8080 | C*15:08 | 0.0003 |
| C*01:50 | 0.0003 | C*03:77 | 0.0006 | C*07:04 | 0.8856 | C*08:06 | 0.0109 | C*15:09 | 0.0003 |
| C*01:54 | 0.0003 | C*03:81 | 0.0006 | C*07:06 | 0.8109 | C*08:16 | 0.0003 | C*15:11 | 0.0121 |
| C*02:02 | 0.7244 | C*03:85 | 0.0015 | C*07:10 | 0.0003 | C*08:20 | 0.0009 | C*15:12 | 0.0009 |
| C*02:10 | 0.0018 | C*03:86 | 0.0003 | C*07:123 | 0.0003 | C*08:21 | 0.0009 | C*15:13 | 0.0165 |
| C*03:02 | 5.8464 | C*03:94 | 0.0003 | C*07:13 | 0.0003 | C*08:22 | 0.9453 | C*15:17 | 0.0009 |
| C*03:03 | 7.1176 | C*03:98 | 0.0006 | C*07:137 | 0.0006 | C*08:24 | 0.0038 | C*15:21 | 0.0009 |
| C*03:04 | 9.9568 | C*04:01 | 5.9758 | C*07:138 | 0.0003 | C*08:27 | 0.0009 | C*15:26 | 0.0047 |
| C*03:07 | 0.0003 | C*04:03 | 1.0077 | C*07:149 | 0.0006 | C*08:40 | 0.0003 | C*15:29 | 0.0018 |
| C*03:100 | 0.0047 | C*04:06 | 0.0318 | C*07:15 | 0.0009 | C*08:41 | 0.0032 | C*15:39 | 0.0003 |
| C*03:107 | 0.0003 | C*04:08 | 0.0024 | C*07:154 | 0.0141 | C*08:44 | 0.0024 | C*16:01 | 0.0056 |
| C*03:16 | 0.0009 | C*04:10 | 0.0003 | C*07:157 | 0.0003 | C*12:02 | 3.1851 | C*16:02 | 0.2015 |
| C*03:17 | 0.0256 | C*04:35 | 0.0003 | C*07:159 | 0.0006 | C*12:03 | 1.9239 | C*16:04 | 0.0868 |
| C*03:21 | 0.0018 | C*04:69 | 0.0050 | C*07:16 | 0.0038 | C*12:04 | 0.0003 | C*17:01 | 0.0991 |
| C*03:28 | 0.0012 | C*04:70 | 0.0006 | C*07:17 | 0.0003 | C*12:05 | 0.0047 | C*17:02 | 0.0024 |
| C*03:34 | 0.0009 | C*04:81 | 0.0006 | C*07:18 | 0.0212 | C*12:09 | 0.0003 | C*17:03 | 0.0200 |
| C*03:35 | 0.0003 | C*04:82 | 0.1227 | C*07:26 | 0.0044 | C*12:10 | 0.0003 | C*18:02 | 0.0021 |
| C*03:36 | 0.0062 | C*05:01 | 0.8874 | C*07:27 | 0.0038 | C*12:18 | 0.0012 |  |  |
| C*03:38 | 0.0024 | C*05:09 | 0.0003 | C*07:39 | 0.0003 | C*14:02 | 4.2390 |  |  |
| C*03:39 | 0.0006 | C*05:30 | 0.0003 | C*07:40 | 0.0003 | C*14:03 | 1.0527 |  |  |
| C*03:40 | 0.0003 | C*06:02 | 8.9282 | C*07:43 | 0.0147 | C*14:12 | 0.0003 |  |  |
