# Supplementary material for: High-Resolution Analyses of Human Leukocyte Antigens Allele and Haplotype Frequencies Based on 169,995 Volunteers from the China Bone Marrow Donor Registry Program
Source: PLoS One. 2015 Sep 30;10(9):e0139485. doi: 10.1371/journal.pone.0139485 (PMC4589403; doi:10.1371/journal.pone.0139485)
Supplement: S4 Table — (DOCX) [file pone.0139485.s004.docx]

**Supporting information**

**S4 Table.**  HLA-DRB1 allele frequencies among the 169,995 CMDP registry donors

| Allele | Freq (%) | Allele | Freq (%) | Allele | Freq (%) | Allele | Freq (%) | Allele | Freq (%) |
| --- | --- | --- | --- | --- | --- | --- | --- | --- | --- |
| DRB1*01:01 | 2.1036 | DRB1*04:86 | 0.0003 | DRB1*11:101 | 0.0012 | DRB1*13:07 | 0.0512 | DRB1*14:45 | 0.0003 |
| DRB1*01:02 | 0.2291 | DRB1*04:88 | 0.0003 | DRB1*11:11 | 0.0071 | DRB1*13:100 | 0.0003 | DRB1*14:49 | 0.0012 |
| DRB1*01:03 | 0.0003 | DRB1*04:93 | 0.0003 | DRB1*11:12 | 0.0003 | DRB1*13:12 | 0.7124 | DRB1*14:54 | 3.2013 |
| DRB1*01:14 | 0.0012 | DRB1*07:01 | 8.9106 | DRB1*11:19 | 0.0009 | DRB1*13:13 | 0.0012 | DRB1*14:61 | 0.0027 |
| DRB1*03:01 | 4.9449 | DRB1*07:13 | 0.0038 | DRB1*11:20 | 0.0021 | DRB1*13:14 | 0.0003 | DRB1*14:63 | 0.0003 |
| DRB1*03:05 | 0.0003 | DRB1*08:01 | 0.0494 | DRB1*11:23 | 0.0012 | DRB1*13:15 | 0.0003 | DRB1*14:77 | 0.0003 |
| DRB1*03:06 | 0.0012 | DRB1*08:02 | 0.6538 | DRB1*11:27 | 0.0006 | DRB1*13:19 | 0.0035 | DRB1*14:96 | 0.0003 |
| DRB1*03:08 | 0.0006 | DRB1*08:03 | 6.1884 | DRB1*11:28 | 0.0100 | DRB1*13:21 | 0.0003 | DRB1*15:01 | 11.6524 |
| DRB1*03:15 | 0.0003 | DRB1*08:04 | 0.0394 | DRB1*11:29 | 0.0003 | DRB1*13:36 | 0.0003 | DRB1*15:02 | 3.0754 |
| DRB1*03:19 | 0.0003 | DRB1*08:09 | 0.1635 | DRB1*11:37 | 0.0006 | DRB1*13:39 | 0.0003 | DRB1*15:03 | 0.0029 |
| DRB1*03:25 | 0.0003 | DRB1*08:12 | 0.0021 | DRB1*11:39 | 0.0012 | DRB1*13:50 | 0.0100 | DRB1*15:04 | 0.2456 |
| DRB1*03:27 | 0.0018 | DRB1*08:14 | 0.0018 | DRB1*11:49 | 0.0003 | DRB1*13:73 | 0.0003 | DRB1*15:06 | 0.0127 |
| DRB1*03:37 | 0.0003 | DRB1*08:19 | 0.0015 | DRB1*11:54 | 0.0015 | DRB1*13:97 | 0.0003 | DRB1*15:07 | 0.0003 |
| DRB1*04:01 | 0.9792 | DRB1*08:32 | 0.0009 | DRB1*11:57 | 0.0006 | DRB1*14:01 | 0.0053 | DRB1*15:11 | 0.0018 |
| DRB1*04:02 | 0.1791 | DRB1*08:33 | 0.0006 | DRB1*11:75 | 0.0009 | DRB1*14:02 | 0.0218 | DRB1*15:31 | 0.0006 |
| DRB1*04:03 | 1.5833 | DRB1*08:35 | 0.0003 | DRB1*12:01 | 3.2895 | DRB1*14:03 | 0.5083 | DRB1*15:47 | 0.0003 |
| DRB1*04:04 | 0.7144 | DRB1*08:36 | 0.0021 | DRB1*12:02 | 8.7044 | DRB1*14:04 | 0.6838 | DRB1*15:49 | 0.0003 |
| DRB1*04:05 | 4.7131 | DRB1*08:37 | 0.0003 | DRB1*12:05 | 0.0068 | DRB1*14:05 | 2.1445 | DRB1*15:51 | 0.0003 |
| DRB1*04:06 | 2.5092 | DRB1*08:41 | 0.0003 | DRB1*12:08 | 0.0079 | DRB1*14:06 | 0.0124 | DRB1*16:01 | 0.0615 |
| DRB1*04:07 | 0.2065 | DRB1*08:45 | 0.0003 | DRB1*12:10 | 0.3265 | DRB1*14:07 | 0.2391 | DRB1*16:02 | 3.1689 |
| DRB1*04:08 | 0.1321 | DRB1*09:01 | 14.3172 | DRB1*12:14 | 0.0006 | DRB1*14:10 | 0.0118 | DRB1*16:05 | 0.0003 |
| DRB1*04:10 | 0.3015 | DRB1*09:04 | 0.0050 | DRB1*12:16 | 0.0006 | DRB1*14:103 | 0.0003 | DRB1*16:09 | 0.0015 |
| DRB1*04:11 | 0.0035 | DRB1*09:10 | 0.0150 | DRB1*12:19 | 0.0003 | DRB1*14:11 | 0.0021 | DRB1*16:10 | 0.0009 |
| DRB1*04:19 | 0.0003 | DRB1*10:01 | 1.5565 | DRB1*12:22 | 0.0003 | DRB1*14:12 | 0.0365 |  |  |
| DRB1*04:38 | 0.0012 | DRB1*10:02 | 0.0003 | DRB1*12:26 | 0.0003 | DRB1*14:18 | 0.0838 |  |  |
| DRB1*04:51 | 0.0003 | DRB1*11:01 | 5.7355 | DRB1*12:27 | 0.0003 | DRB1*14:22 | 0.0035 |  |  |
| DRB1*04:59 | 0.0006 | DRB1*11:03 | 0.0274 | DRB1*13:01 | 1.4633 | DRB1*14:25 | 0.0153 |  |  |
| DRB1*04:68 | 0.0003 | DRB1*11:04 | 0.6044 | DRB1*13:02 | 3.1336 | DRB1*14:33 | 0.0006 |  |  |
| DRB1*04:78 | 0.0003 | DRB1*11:06 | 0.1124 | DRB1*13:03 | 0.0435 | DRB1*14:35 | 0.0003 |  |  |
| DRB1*04:80 | 0.0003 | DRB1*11:08 | 0.0003 | DRB1*13:05 | 0.0059 | DRB1*14:44 | 0.0032 |  |  |
