# Supplementary material for: High-Resolution Analyses of Human Leukocyte Antigens Allele and Haplotype Frequencies Based on 169,995 Volunteers from the China Bone Marrow Donor Registry Program
Source: PLoS One. 2015 Sep 30;10(9):e0139485. doi: 10.1371/journal.pone.0139485 (PMC4589403; doi:10.1371/journal.pone.0139485)
Supplement: S5 Table — (DOCX) [file pone.0139485.s005.docx]

**Supporting information**

**S5 Table.**  HLA-DQB1 allele frequencies among the 169,995 CMDP registry donors

| Allele | Freq (%) | Allele | Freq (%) | Allele | Freq (%) | Allele | Freq (%) | Allele | Freq (%) |
| --- | --- | --- | --- | --- | --- | --- | --- | --- | --- |
| DQB1*02:01 | 4.9325 | DQB1*03:13 | 0.0824 | DQB1*04:01 | 4.4769 | DQB1*06:01 | 10.1830 | DQB1*06:14 | 0.0006 |
| DQB1*02:02 | 7.5741 | DQB1*03:14 | 0.0015 | DQB1*04:02 | 1.2524 | DQB1*06:02 | 7.8552 | DQB1*06:22 | 0.0003 |
| DQB1*02:03 | 0.0003 | DQB1*03:17 | 0.0109 | DQB1*04:03 | 0.0003 | DQB1*06:03 | 1.4956 | DQB1*06:24 | 0.0003 |
| DQB1*02:06 | 0.0003 | DQB1*03:22 | 0.0053 | DQB1*05:01 | 4.6657 | DQB1*06:04 | 1.4571 | DQB1*06:28 | 0.0003 |
| DQB1*03:01 | 21.0942 | DQB1*03:26 | 0.0009 | DQB1*05:02 | 7.2905 | DQB1*06:07 | 0.0088 | DQB1*06:31 | 0.0003 |
| DQB1*03:02 | 5.7472 | DQB1*03:27 | 0.0027 | DQB1*05:03 | 4.2495 | DQB1*06:08 | 0.0003 | DQB1*06:41 | 0.0032 |
| DQB1*03:03 | 15.7034 | DQB1*03:29 | 0.0029 | DQB1*05:04 | 0.0124 | DQB1*06:09 | 1.6465 | DQB1*06:42 | 0.0003 |
| DQB1*03:04 | 0.0318 | DQB1*03:30 | 0.0006 | DQB1*05:05 | 0.0006 | DQB1*06:10 | 0.1062 |  |  |
| DQB1*03:05 | 0.0879 | DQB1*03:34 | 0.0003 | DQB1*05:08 | 0.0009 | DQB1*06:11 | 0.0015 |  |  |
| DQB1*03:12 | 0.0003 | DQB1*03:38 | 0.0003 | DQB1*05:10 | 0.0115 | DQB1*06:12 | 0.0003 |  |  |
