# Supplementary material for: High-Resolution Analyses of Human Leukocyte Antigens Allele and Haplotype Frequencies Based on 169,995 Volunteers from the China Bone Marrow Donor Registry Program
Source: PLoS One. 2015 Sep 30;10(9):e0139485. doi: 10.1371/journal.pone.0139485 (PMC4589403; doi:10.1371/journal.pone.0139485)
Supplement: S6 Table — (DOCX) [file pone.0139485.s006.docx]

**Supporting information**

**S6 Table. The pairwise linkage disequilibria for the c**ommon (F-est.>1‰) HLA two-locus haplotypes among the 169,995 CMDP registry donors.

| HLA-A-C | | | | | HLA-C-B | | | | | HLA-DRB1-DQB1 | | | | |
| --- | --- | --- | --- | --- | --- | --- | --- | --- | --- | --- | --- | --- | --- | --- |
| A | C | F-est (‰) | F-exp (‰) | p value (LD) | C | B | F-est  (‰) | F-exp  (‰) | p value (LD) | DRB1 | DQB1 | F-est  (‰) | F-exp  (‰) | p value (LD) |
| 02:07 | 01:02 | 56.2810 | 13.2301 | * | 01:02 | 46:01 | 90.9520 | 15.9016 | * | 09:01 | 03:03 | 138.4584 | 22.4829 | * |
| 11:01 | 07:02 | 48.0546 | 32.0357 | * | 03:02 | 58:01 | 57.0082 | 3.4314 | * | 12:02 | 03:01 | 81.5595 | 18.3611 | * |
| 30:01 | 06:02 | 45.8502 | 4.8516 | * | 06:02 | 13:02 | 56.5344 | 5.1507 | * | 15:01 | 06:02 | 76.1416 | 9.1532 | * |
| 33:03 | 03:02 | 44.9588 | 4.6368 | * | 07:02 | 40:01 | 47.6447 | 15.0851 | * | 07:01 | 02:02 | 74.6966 | 6.7489 | * |
| 11:01 | 01:02 | 31.5274 | 32.8951 | * | 03:04 | 13:01 | 44.0975 | 4.9914 | * | 08:03 | 06:01 | 57.8060 | 6.3016 | * |
| 11:01 | 03:04 | 31.4750 | 21.0515 | * | 14:02 | 51:01 | 39.2192 | 2.3892 | * | 11:01 | 03:01 | 53.7525 | 12.0985 | * |
| 11:01 | 08:01 | 30.4845 | 17.8918 | * | 08:01 | 15:02 | 34.0352 | 2.9995 | * | 03:01 | 02:01 | 48.8513 | 2.4390 | * |
| 24:02 | 01:02 | 25.1181 | 24.2224 | # | 03:04 | 40:01 | 32.7183 | 9.9128 | * | 04:05 | 04:01 | 43.3878 | 2.1100 | * |
| 24:02 | 03:04 | 24.4520 | 15.5014 | * | 01:02 | 54:01 | 27.8765 | 4.7281 | * | 16:02 | 05:02 | 31.0764 | 2.3103 | * |
| 24:02 | 07:02 | 23.9744 | 23.5896 | 0.15 | 07:02 | 38:02 | 25.0962 | 3.9592 | * | 12:01 | 03:01 | 30.1136 | 6.9389 | * |
| 02:01 | 07:02 | 20.1926 | 18.6214 | * | 08:01 | 40:06 | 22.6509 | 2.7351 | * | 04:06 | 03:02 | 24.7123 | 1.4421 | * |
| 02:01 | 03:03 | 18.4313 | 8.7473 | * | 12:02 | 52:01 | 21.2140 | 0.9091 | * | 15:01 | 06:01 | 23.8793 | 11.8656 | * |
| 01:01 | 06:02 | 17.6409 | 3.0388 | * | 07:02 | 07:02 | 20.5020 | 3.3095 | * | 14:54 | 05:02 | 21.1896 | 2.3339 | * |
| 02:03 | 07:02 | 16.4587 | 5.2975 | * | 03:03 | 15:11 | 17.2743 | 1.3036 | * | 01:01 | 05:01 | 20.7260 | 0.9815 | * |
| 02:01 | 03:04 | 16.1802 | 12.2366 | * | 03:03 | 35:01 | 16.5727 | 2.0302 | * | 14:05 | 05:03 | 20.3566 | 0.9113 | * |
| 24:02 | 08:01 | 15.2163 | 13.1747 | * | 03:03 | 15:01 | 16.2786 | 3.4994 | * | 15:02 | 06:01 | 17.6637 | 3.1316 | * |
| 11:01 | 04:01 | 15.0614 | 12.6345 | * | 04:01 | 15:01 | 16.2509 | 2.9381 | * | 13:02 | 06:09 | 16.3540 | 0.5160 | * |
| 02:01 | 01:02 | 14.9840 | 19.1209 | * | 07:02 | 39:01 | 15.2918 | 2.7034 | * | 10:01 | 05:01 | 15.4070 | 0.7262 | * |
| 24:02 | 03:03 | 14.8833 | 11.0811 | * | 06:02 | 37:01 | 13.0302 | 1.2024 | * | 04:03 | 03:02 | 14.7098 | 0.9099 | * |
| 24:02 | 04:01 | 11.3818 | 9.3035 | * | 01:02 | 55:02 | 11.9909 | 3.8142 | * | 13:01 | 06:03 | 14.4748 | 0.2189 | * |
| 02:01 | 08:01 | 11.0774 | 10.4000 | # | 06:02 | 57:01 | 10.4281 | 0.9750 | * | 13:02 | 06:04 | 14.4480 | 0.4566 | * |
| 11:01 | 14:02 | 10.8745 | 8.9624 | * | 14:03 | 44:03 | 10.3606 | 0.2768 | * | 07:01 | 03:03 | 12.9044 | 13.9926 | * |
| 11:01 | 03:03 | 10.7405 | 15.0486 | * | 15:02 | 51:01 | 10.3520 | 1.9050 | * | 14:54 | 05:03 | 9.3030 | 1.3604 | * |
| 24:02 | 14:02 | 9.7806 | 6.5995 | * | 15:02 | 51:02 | 9.2494 | 0.3571 | * | 04:01 | 03:01 | 7.6134 | 2.0654 | * |
| 02:06 | 08:01 | 8.9535 | 4.4000 | * | 08:01 | 48:01 | 8.5013 | 2.0830 | * | 15:01 | 05:02 | 7.5224 | 8.4952 | * |
| 33:03 | 14:03 | 8.6534 | 0.8349 | * | 03:04 | 40:02 | 8.3705 | 1.9214 | * | 15:02 | 05:01 | 7.4633 | 1.4349 | * |
| 02:06 | 03:03 | 8.1815 | 3.7008 | * | 04:01 | 35:01 | 8.3540 | 1.7045 | * | 13:12 | 03:01 | 6.9137 | 1.5027 | * |
| 02:01 | 15:02 | 6.5913 | 4.1537 | * | 05:01 | 44:02 | 8.1386 | 0.0867 | * | 14:04 | 05:03 | 6.7308 | 0.2906 | * |
| 11:01 | 12:02 | 6.4658 | 6.7342 | 0.09 | 07:02 | 08:01 | 8.0703 | 1.3062 | * | 04:04 | 03:02 | 6.6521 | 0.4106 | * |
| 29:01 | 15:05 | 6.2581 | 0.0626 | * | 07:06 | 44:03 | 7.9887 | 0.2132 | * | 11:04 | 03:01 | 5.8441 | 1.2750 | * |
| 24:02 | 15:02 | 6.1343 | 5.2619 | * | 01:02 | 15:01 | 7.8452 | 7.6495 | 0.18 | 08:02 | 04:02 | 5.5799 | 0.0819 | * |
| 02:07 | 07:02 | 6.0215 | 12.8844 | * | 04:01 | 15:27 | 7.5858 | 0.4837 | * | 14:03 | 03:01 | 4.9405 | 1.0721 | * |
| 33:03 | 07:06 | 5.9267 | 0.6431 | * | 12:02 | 27:04 | 7.5013 | 0.3007 | * | 15:02 | 05:02 | 3.9615 | 2.2421 | * |
| 26:01 | 07:02 | 5.8872 | 4.2378 | * | 08:03 | 48:01 | 7.4609 | 0.1989 | * | 15:01 | 03:01 | 3.6673 | 24.5797 | * |
| 02:01 | 04:01 | 5.8556 | 7.3441 | * | 15:05 | 07:05 | 7.2442 | 0.0544 | * | 12:02 | 05:02 | 3.6531 | 6.3459 | * |
| 02:06 | 01:02 | 5.6851 | 8.0897 | * | 07:02 | 67:01 | 7.2040 | 1.1315 | * | 12:10 | 03:01 | 3.1770 | 0.6887 | * |
| 02:01 | 14:02 | 5.6369 | 5.2096 | # | 04:01 | 44:03 | 7.0552 | 1.5713 | * | 08:03 | 03:01 | 3.0428 | 13.0539 | * |
| 31:01 | 15:02 | 5.6357 | 1.1149 | * | 08:01 | 15:18 | 6.7052 | 1.1385 | * | 04:10 | 04:02 | 2.9414 | 0.0378 | * |
| 11:01 | 15:02 | 5.5817 | 7.1459 | * | 02:02 | 27:05 | 6.4956 | 0.0555 | * | 15:01 | 05:03 | 2.6653 | 4.9517 | * |
| 03:01 | 07:02 | 5.5634 | 4.5475 | * | 07:04 | 15:18 | 6.2666 | 0.1191 | * | 15:04 | 05:02 | 2.4105 | 0.1791 | * |
| 02:06 | 14:02 | 5.5551 | 2.2041 | * | 07:02 | 52:01 | 6.2434 | 4.3247 | * | 01:02 | 05:01 | 2.2727 | 0.1069 | * |
| 02:06 | 07:02 | 5.2719 | 7.8783 | * | 01:03 | 46:01 | 5.9492 | 0.6334 | * | 04:01 | 03:02 | 1.9468 | 0.5627 | * |
| 31:01 | 03:03 | 4.9786 | 2.3478 | * | 06:02 | 50:01 | 5.9171 | 0.5536 | * | 04:05 | 03:02 | 1.7676 | 2.7087 | * |
| 02:06 | 03:04 | 4.8867 | 5.1771 | # | 04:01 | 35:03 | 5.8995 | 0.6609 | * | 04:02 | 03:02 | 1.7566 | 0.1029 | * |
| 11:02 | 12:02 | 4.7752 | 0.6105 | * | 03:03 | 40:02 | 5.4100 | 1.3735 | * | 08:09 | 04:02 | 1.6203 | 0.0205 | * |
| 32:01 | 04:01 | 4.4500 | 0.7644 | * | 15:02 | 40:01 | 5.3741 | 3.3649 | * | 09:01 | 03:02 | 1.4476 | 8.2284 | * |
| 02:01 | 06:02 | 4.4245 | 10.9726 | * | 04:03 | 15:25 | 5.1509 | 0.0629 | * | 09:01 | 03:01 | 1.4256 | 30.2009 | * |
| 32:01 | 12:02 | 4.4075 | 0.4074 | * | 12:03 | 38:01 | 4.7539 | 0.0940 | * | 14:07 | 05:03 | 1.3134 | 0.1016 | * |
| 33:03 | 07:02 | 4.3178 | 12.0172 | * | 12:03 | 35:03 | 4.4930 | 0.2128 | * | 11:01 | 03:03 | 1.2538 | 9.0066 | * |
| 11:01 | 12:03 | 4.2343 | 4.0677 | 0.14 | 12:03 | 55:02 | 4.4656 | 0.4716 | * | 04:07 | 03:02 | 1.1779 | 0.1187 | * |
| 03:01 | 05:01 | 4.2207 | 0.2663 | * | 08:22 | 40:06 | 4.1137 | 0.3055 | * | 11:06 | 03:01 | 1.0877 | 0.2370 | * |
| 24:02 | 06:02 | 3.9528 | 13.9000 | * | 08:22 | 48:01 | 3.9959 | 0.2327 | * | 14:07 | 05:02 | 1.0392 | 0.1743 | * |
| 02:07 | 03:04 | 3.8752 | 8.4667 | * | 04:01 | 40:01 | 3.6061 | 5.9494 | * | 15:01 | 06:10 | 1.0390 | 0.1237 | * |
| 02:03 | 01:02 | 3.8596 | 5.4397 | * | 08:01 | 15:01 | 3.2123 | 4.1606 | * | 15:02 | 06:02 | 1.0023 | 2.4158 | * |
| 11:01 | 06:02 | 3.7335 | 18.8769 | * | 07:02 | 15:01 | 3.0403 | 7.4496 | * |  |  |  |  |  |
| 26:01 | 03:04 | 3.7092 | 2.7848 | * | 07:02 | 13:01 | 2.7423 | 7.5958 | * |  |  |  |  |  |
| 11:01 | 03:02 | 3.6407 | 12.3609 | * | 03:04 | 40:06 | 2.7213 | 3.2182 | * |  |  |  |  |  |
| 02:01 | 08:22 | 3.5771 | 1.1618 | * | 04:01 | 35:05 | 2.5925 | 0.1580 | * |  |  |  |  |  |
| 02:03 | 03:04 | 3.5217 | 3.4812 | 0.39 | 03:03 | 55:02 | 2.5783 | 1.7449 | * |  |  |  |  |  |
| 31:01 | 03:04 | 3.2834 | 3.2844 | 0.50 | 08:02 | 14:02 | 2.5721 | 0.0079 | * |  |  |  |  |  |
| 02:07 | 01:03 | 3.2511 | 0.5270 | * | 03:03 | 15:07 | 2.4984 | 0.2263 | * |  |  |  |  |  |
| 02:03 | 04:03 | 3.2468 | 0.3523 | * | 01:02 | 56:01 | 2.4858 | 0.8059 | * |  |  |  |  |  |
| 11:02 | 07:02 | 3.2275 | 2.9044 | # | 01:02 | 40:01 | 2.2397 | 15.4898 | * |  |  |  |  |  |
| 26:01 | 01:02 | 3.1438 | 4.3515 | * | 15:02 | 40:02 | 2.2152 | 0.6522 | * |  |  |  |  |  |
| 31:01 | 04:01 | 3.1005 | 1.9712 | * | 07:02 | 56:01 | 2.1957 | 0.7848 | * |  |  |  |  |  |
| 26:01 | 03:03 | 3.0822 | 1.9907 | * | 12:03 | 15:32 | 2.0618 | 0.0431 | * |  |  |  |  |  |
| 31:01 | 01:02 | 3.0760 | 5.1321 | * | 03:03 | 40:01 | 1.9906 | 7.0862 | * |  |  |  |  |  |
| 02:10 | 08:01 | 3.0585 | 0.3514 | * | 03:04 | 40:03 | 1.9767 | 0.2202 | * |  |  |  |  |  |
| 31:01 | 07:02 | 3.0292 | 4.9981 | * | 04:01 | 35:02 | 1.9000 | 0.1178 | * |  |  |  |  |  |
| 33:03 | 01:02 | 2.9516 | 12.3396 | * | 03:03 | 15:12 | 1.8690 | 0.1897 | * |  |  |  |  |  |
| 02:07 | 08:01 | 2.9418 | 7.1959 | * | 07:01 | 15:17 | 1.8240 | 0.0105 | * |  |  |  |  |  |
| 01:01 | 07:02 | 2.9163 | 5.1572 | * | 07:02 | 39:05 | 1.7141 | 0.2701 | * |  |  |  |  |  |
| 24:02 | 12:02 | 2.8968 | 4.9588 | * | 12:03 | 18:01 | 1.6752 | 0.0768 | * |  |  |  |  |  |
| 03:01 | 04:01 | 2.8333 | 1.7935 | * | 04:03 | 40:01 | 1.6674 | 1.0032 | * |  |  |  |  |  |
| 24:02 | 08:03 | 2.7958 | 1.2579 | * | 04:01 | 15:05 | 1.5859 | 0.0986 | * |  |  |  |  |  |
| 02:01 | 07:04 | 2.7908 | 1.0884 | * | 07:01 | 49:01 | 1.4896 | 0.0085 | * |  |  |  |  |  |
| 11:02 | 01:02 | 2.7856 | 2.9823 | 0.07 | 08:01 | 48:03 | 1.4712 | 0.1357 | * |  |  |  |  |  |
| 03:01 | 02:02 | 2.7521 | 0.2174 | * | 07:01 | 18:01 | 1.3822 | 0.0222 | * |  |  |  |  |  |
| 31:01 | 14:02 | 2.7021 | 1.3983 | * | 01:02 | 51:01 | 1.3732 | 8.7692 | * |  |  |  |  |  |
| 03:01 | 12:03 | 2.7012 | 0.5774 | * | 08:01 | 35:01 | 1.3683 | 2.4138 | * |  |  |  |  |  |
| 02:01 | 12:02 | 2.6511 | 3.9144 | * | 07:02 | 40:02 | 1.3649 | 2.9240 | * |  |  |  |  |  |
| 02:05 | 06:02 | 2.6477 | 0.2988 | * | 16:02 | 51:01 | 1.3282 | 0.1136 | * |  |  |  |  |  |
| 02:06 | 04:01 | 2.5717 | 3.1071 | * | 15:02 | 27:07 | 1.3212 | 0.0473 | * |  |  |  |  |  |
| 24:02 | 03:02 | 2.4970 | 9.1020 | * | 07:02 | 55:02 | 1.2597 | 3.7146 | * |  |  |  |  |  |
| 03:01 | 12:02 | 2.4919 | 0.9559 | * | 03:04 | 15:01 | 1.2488 | 4.8954 | * |  |  |  |  |  |
| 26:01 | 12:03 | 2.4404 | 0.5381 | * | 07:02 | 46:01 | 1.1782 | 15.4862 | * |  |  |  |  |  |
| 24:02 | 12:03 | 2.4348 | 2.9952 | * | 07:02 | 51:01 | 1.1561 | 8.5401 | * |  |  |  |  |  |
| 31:01 | 08:01 | 2.4282 | 2.7914 | # | 06:02 | 45:01 | 1.0856 | 0.1003 | * |  |  |  |  |  |
| 02:07 | 03:03 | 2.3619 | 6.0524 | * | 04:03 | 55:02 | 1.0137 | 0.2470 | * |  |  |  |  |  |
| 24:02 | 04:03 | 2.3447 | 1.5688 | * |  |  |  |  |  |  |  |  |  |  |
| 11:01 | 04:03 | 2.1713 | 2.1305 | 0.36 |  |  |  |  |  |  |  |  |  |  |
| 02:01 | 08:03 | 2.1594 | 0.9930 | * |  |  |  |  |  |  |  |  |  |  |
| 02:03 | 08:01 | 1.9613 | 2.9587 | * |  |  |  |  |  |  |  |  |  |  |
| 02:06 | 15:02 | 1.9560 | 1.7574 | # |  |  |  |  |  |  |  |  |  |  |
| 02:07 | 04:01 | 1.9211 | 5.0815 | * |  |  |  |  |  |  |  |  |  |  |
| 11:02 | 03:04 | 1.9206 | 1.9085 | 0.45 |  |  |  |  |  |  |  |  |  |  |
| 01:01 | 07:01 | 1.8323 | 0.1892 | * |  |  |  |  |  |  |  |  |  |  |
| 33:03 | 03:04 | 1.8287 | 7.8968 | * |  |  |  |  |  |  |  |  |  |  |
| 02:07 | 14:02 | 1.7729 | 3.6046 | * |  |  |  |  |  |  |  |  |  |  |
| 01:01 | 12:02 | 1.7662 | 1.0841 | * |  |  |  |  |  |  |  |  |  |  |
| 24:02 | 08:22 | 1.7612 | 1.4717 | * |  |  |  |  |  |  |  |  |  |  |
| 02:01 | 03:02 | 1.7558 | 7.1850 | * |  |  |  |  |  |  |  |  |  |  |
| 01:01 | 04:01 | 1.7201 | 2.0339 | # |  |  |  |  |  |  |  |  |  |  |
| 11:02 | 08:01 | 1.7123 | 1.6221 | 0.18 |  |  |  |  |  |  |  |  |  |  |
| 26:01 | 04:01 | 1.6492 | 1.6713 | 0.41 |  |  |  |  |  |  |  |  |  |  |
| 02:06 | 08:22 | 1.6387 | 0.4915 | * |  |  |  |  |  |  |  |  |  |  |
| 03:01 | 15:02 | 1.6349 | 1.0144 | * |  |  |  |  |  |  |  |  |  |  |
| 26:01 | 08:01 | 1.6124 | 2.3668 | * |  |  |  |  |  |  |  |  |  |  |
| 02:06 | 06:02 | 1.5607 | 4.6423 | * |  |  |  |  |  |  |  |  |  |  |
| 33:03 | 04:01 | 1.5245 | 4.7394 | * |  |  |  |  |  |  |  |  |  |  |
| 33:03 | 08:01 | 1.4731 | 6.7116 | * |  |  |  |  |  |  |  |  |  |  |
| 33:01 | 08:02 | 1.4519 | 0.0047 | * |  |  |  |  |  |  |  |  |  |  |
| 02:03 | 12:03 | 1.4470 | 0.6726 | * |  |  |  |  |  |  |  |  |  |  |
| 26:01 | 15:02 | 1.4258 | 0.9453 | * |  |  |  |  |  |  |  |  |  |  |
| 24:07 | 04:01 | 1.4153 | 0.1288 | * |  |  |  |  |  |  |  |  |  |  |
| 33:03 | 06:02 | 1.4088 | 7.0811 | * |  |  |  |  |  |  |  |  |  |  |
| 02:01 | 12:03 | 1.3670 | 2.3644 | * |  |  |  |  |  |  |  |  |  |  |
| 03:01 | 06:02 | 1.3284 | 2.6796 | * |  |  |  |  |  |  |  |  |  |  |
| 33:03 | 03:03 | 1.2972 | 5.6450 | * |  |  |  |  |  |  |  |  |  |  |
| 68:01 | 04:01 | 1.2645 | 0.4438 | * |  |  |  |  |  |  |  |  |  |  |
| 03:01 | 01:02 | 1.2537 | 4.6695 | * |  |  |  |  |  |  |  |  |  |  |
| 02:01 | 05:01 | 1.2304 | 1.0906 | # |  |  |  |  |  |  |  |  |  |  |
| 11:02 | 04:01 | 1.2294 | 1.1454 | 0.15 |  |  |  |  |  |  |  |  |  |  |
| 01:01 | 01:02 | 1.2283 | 5.2955 | * |  |  |  |  |  |  |  |  |  |  |
| 24:02 | 07:04 | 1.2246 | 1.3788 | # |  |  |  |  |  |  |  |  |  |  |
| 30:01 | 07:02 | 1.1904 | 8.2335 | * |  |  |  |  |  |  |  |  |  |  |
| 33:03 | 12:03 | 1.1284 | 1.5259 | * |  |  |  |  |  |  |  |  |  |  |
| 02:07 | 15:02 | 1.1204 | 2.8740 | * |  |  |  |  |  |  |  |  |  |  |
| 02:07 | 03:02 | 1.1037 | 4.9714 | * |  |  |  |  |  |  |  |  |  |  |
| 26:01 | 14:02 | 1.0942 | 1.1856 | 0.14 |  |  |  |  |  |  |  |  |  |  |
| 11:01 | 07:04 | 1.0926 | 1.8724 | * |  |  |  |  |  |  |  |  |  |  |
| 23:01 | 04:01 | 1.0704 | 0.1610 | * |  |  |  |  |  |  |  |  |  |  |
| 30:01 | 01:02 | 1.0623 | 8.4544 | * |  |  |  |  |  |  |  |  |  |  |
| 03:01 | 03:03 | 1.0094 | 2.1362 | * |  |  |  |  |  |  |  |  |  |  |

F-est: estimated frequency;F-exp: expected frequency; the p value is for the pairwise LD calculation: *p<0.001, #p<0.05(Chi-square test).
