# Supplementary material for: High-Resolution Analyses of Human Leukocyte Antigens Allele and Haplotype Frequencies Based on 169,995 Volunteers from the China Bone Marrow Donor Registry Program
Source: PLoS One. 2015 Sep 30;10(9):e0139485. doi: 10.1371/journal.pone.0139485 (PMC4589403; doi:10.1371/journal.pone.0139485)
Supplement: S7 Table — (DOCX) [file pone.0139485.s007.docx]

**Supporting information**

**S7 Table.** Common (freq.>1‰) HLA three-locus haplotypes among the 169,995 CMDP registry donors

| HLA-A-C-B | | | | HLA-A-B-DRB1 | | | |
| --- | --- | --- | --- | --- | --- | --- | --- |
| HLA-A | HLA-C | HLA-B | Freq (‰) | HLA-A | HLA-B | HLA-DRB1 | Freq (‰) |
| 02:07 | 01:02 | 46:01 | 54.4084 | 30:01 | 13:02 | 07:01 | 36.8742 |
| 30:01 | 06:02 | 13:02 | 45.3716 | 02:07 | 46:01 | 09:01 | 26.6153 |
| 33:03 | 03:02 | 58:01 | 44.3353 | 33:03 | 58:01 | 03:01 | 23.7658 |
| 11:01 | 07:02 | 40:01 | 22.8550 | 11:01 | 15:02 | 12:02 | 11.7517 |
| 11:01 | 08:01 | 15:02 | 22.0362 | 33:03 | 58:01 | 13:02 | 10.6254 |
| 11:01 | 03:04 | 13:01 | 18.3770 | 02:07 | 46:01 | 08:03 | 9.6282 |
| 11:01 | 01:02 | 46:01 | 12.6275 | 33:03 | 44:03 | 13:02 | 7.4092 |
| 02:03 | 07:02 | 38:02 | 12.3285 | 11:01 | 13:01 | 15:01 | 7.3378 |
| 24:02 | 01:02 | 54:01 | 11.2345 | 11:01 | 46:01 | 09:01 | 6.8425 |
| 24:02 | 03:04 | 40:01 | 10.9298 | 01:01 | 37:01 | 10:01 | 6.6792 |
| 11:01 | 14:02 | 51:01 | 10.0684 | 02:01 | 13:01 | 12:02 | 6.1662 |
| 11:01 | 03:04 | 40:01 | 9.7129 | 11:01 | 15:01 | 04:06 | 5.7222 |
| 24:02 | 14:02 | 51:01 | 9.2264 | 11:01 | 40:01 | 09:01 | 5.6689 |
| 01:01 | 06:02 | 37:01 | 9.1230 | 11:01 | 15:02 | 15:01 | 4.9870 |
| 02:01 | 03:03 | 15:11 | 9.1106 | 11:01 | 40:01 | 08:03 | 4.9521 |
| 02:01 | 03:04 | 13:01 | 8.8053 | 11:01 | 13:01 | 12:02 | 4.8652 |
| 33:03 | 14:03 | 44:03 | 8.6005 | 33:03 | 44:03 | 07:01 | 4.8533 |
| 24:02 | 07:02 | 40:01 | 8.3533 | 01:01 | 57:01 | 07:01 | 4.8465 |
| 11:01 | 04:01 | 15:01 | 8.1900 | 24:02 | 54:01 | 04:05 | 4.7233 |
| 24:02 | 03:04 | 13:01 | 7.5380 | 11:01 | 40:01 | 15:01 | 4.6488 |
| 24:02 | 01:02 | 46:01 | 7.4066 | 02:07 | 46:01 | 14:54 | 4.2404 |
| 01:01 | 06:02 | 57:01 | 6.9961 | 11:01 | 40:01 | 11:01 | 3.9881 |
| 29:01 | 15:05 | 07:05 | 6.2100 | 24:02 | 46:01 | 09:01 | 3.9195 |
| 24:02 | 08:01 | 40:06 | 5.9430 | 02:03 | 38:02 | 16:02 | 3.7576 |
| 33:03 | 07:06 | 44:03 | 5.9035 | 24:02 | 40:01 | 15:01 | 3.7066 |
| 11:01 | 01:02 | 54:01 | 5.8535 | 02:01 | 15:11 | 09:01 | 3.6936 |
| 02:06 | 14:02 | 51:01 | 5.4701 | 24:02 | 40:01 | 09:01 | 3.6039 |
| 02:01 | 01:02 | 46:01 | 5.3761 | 24:02 | 40:06 | 09:01 | 3.5416 |
| 11:01 | 07:02 | 38:02 | 5.3396 | 24:02 | 51:01 | 09:01 | 3.4837 |
| 02:01 | 14:02 | 51:01 | 5.2724 | 11:01 | 51:01 | 09:01 | 3.4800 |
| 03:01 | 07:02 | 07:02 | 5.1980 | 24:02 | 40:01 | 11:01 | 3.2914 |
| 02:01 | 07:02 | 40:01 | 5.1222 | 32:01 | 52:01 | 15:02 | 3.1998 |
| 11:01 | 07:02 | 39:01 | 5.0968 | 03:01 | 07:02 | 15:01 | 2.9788 |
| 11:01 | 01:02 | 55:02 | 4.9147 | 11:01 | 40:01 | 12:02 | 2.9788 |
| 02:06 | 08:01 | 40:06 | 4.6142 | 11:01 | 07:02 | 01:01 | 2.9738 |
| 11:01 | 12:02 | 52:01 | 4.6123 | 02:01 | 46:01 | 09:01 | 2.9176 |
| 32:01 | 12:02 | 52:01 | 4.3919 | 11:01 | 54:01 | 04:05 | 2.7673 |
| 11:01 | 07:02 | 07:02 | 4.0894 | 02:07 | 46:01 | 15:01 | 2.7466 |
| 02:01 | 01:02 | 54:01 | 4.0886 | 24:02 | 13:01 | 12:02 | 2.7406 |
| 03:01 | 05:01 | 44:02 | 4.0744 | 11:01 | 13:01 | 16:02 | 2.6007 |
| 24:02 | 03:03 | 35:01 | 4.0096 | 02:01 | 15:01 | 15:01 | 2.5786 |
| 02:01 | 07:02 | 67:01 | 3.9813 | 02:07 | 46:01 | 12:02 | 2.5690 |
| 11:02 | 12:02 | 27:04 | 3.9615 | 11:01 | 52:01 | 15:02 | 2.5666 |
| 02:01 | 03:04 | 40:01 | 3.8221 | 24:02 | 40:01 | 08:03 | 2.5232 |
| 24:02 | 07:02 | 07:02 | 3.4991 | 11:01 | 40:01 | 04:05 | 2.5207 |
| 02:01 | 03:03 | 15:01 | 3.4585 | 03:01 | 44:02 | 13:01 | 2.4483 |
| 11:01 | 03:02 | 58:01 | 3.4012 | 02:01 | 40:01 | 09:01 | 2.3809 |
| 24:02 | 04:01 | 15:27 | 3.2787 | 11:01 | 40:01 | 12:01 | 2.3239 |
| 32:01 | 04:01 | 44:03 | 3.2596 | 02:01 | 40:06 | 09:01 | 2.3099 |
| 31:01 | 15:02 | 51:02 | 3.2379 | 32:01 | 44:03 | 07:01 | 2.2511 |
| 24:02 | 08:01 | 15:02 | 3.1662 | 02:03 | 38:02 | 08:03 | 2.1412 |
| 02:07 | 01:03 | 46:01 | 3.1634 | 24:02 | 13:01 | 15:01 | 2.1408 |
| 02:01 | 03:03 | 35:01 | 3.1522 | 02:01 | 51:01 | 09:01 | 2.1262 |
| 31:01 | 03:03 | 15:01 | 3.1252 | 11:01 | 58:01 | 03:01 | 2.0999 |
| 11:01 | 08:01 | 40:06 | 3.0925 | 02:05 | 50:01 | 07:01 | 2.0905 |
| 24:02 | 04:01 | 15:01 | 2.9712 | 31:01 | 15:01 | 15:01 | 2.0660 |
| 02:10 | 08:01 | 40:06 | 2.9500 | 11:01 | 46:01 | 08:03 | 2.0434 |
| 24:02 | 07:02 | 39:01 | 2.8922 | 02:03 | 38:02 | 15:02 | 1.9006 |
| 02:06 | 03:03 | 15:11 | 2.8030 | 02:07 | 46:01 | 04:05 | 1.8813 |
| 11:01 | 03:03 | 35:01 | 2.7610 | 02:07 | 46:01 | 11:01 | 1.8679 |
| 24:02 | 03:04 | 40:02 | 2.7138 | 24:02 | 40:01 | 12:02 | 1.8678 |
| 03:01 | 02:02 | 27:05 | 2.6895 | 02:01 | 13:02 | 07:01 | 1.8616 |
| 24:02 | 03:03 | 15:01 | 2.6763 | 24:02 | 15:27 | 04:06 | 1.8495 |
| 02:01 | 08:01 | 40:06 | 2.6718 | 11:01 | 39:01 | 08:03 | 1.8459 |
| 02:06 | 03:03 | 35:01 | 2.6513 | 24:02 | 15:02 | 12:02 | 1.8272 |
| 02:05 | 06:02 | 50:01 | 2.6382 | 02:01 | 40:01 | 15:01 | 1.8253 |
| 24:02 | 08:03 | 48:01 | 2.6300 | 02:06 | 40:06 | 09:01 | 1.8093 |
| 11:01 | 03:03 | 15:01 | 2.6205 | 24:02 | 15:01 | 15:01 | 1.7810 |
| 03:01 | 12:02 | 52:01 | 2.4780 | 24:02 | 48:01 | 15:01 | 1.7489 |
| 02:07 | 07:02 | 40:01 | 2.4625 | 24:02 | 35:01 | 15:01 | 1.7424 |
| 24:02 | 08:01 | 48:01 | 2.4514 | 02:01 | 40:01 | 11:01 | 1.7274 |
| 31:01 | 14:02 | 51:01 | 2.4429 | 11:01 | 15:01 | 15:01 | 1.7269 |
| 24:02 | 03:02 | 58:01 | 2.3888 | 24:02 | 07:02 | 15:01 | 1.7251 |
| 02:01 | 07:04 | 15:18 | 2.3663 | 24:02 | 40:01 | 04:05 | 1.6977 |
| 26:01 | 12:03 | 38:01 | 2.3129 | 02:01 | 15:01 | 09:01 | 1.6877 |
| 02:01 | 07:02 | 39:01 | 2.2478 | 29:01 | 07:05 | 10:01 | 1.6728 |
| 24:02 | 07:02 | 38:02 | 2.2342 | 24:02 | 15:01 | 04:06 | 1.6724 |
| 02:01 | 07:02 | 07:02 | 2.2330 | 11:01 | 40:01 | 16:02 | 1.6722 |
| 02:01 | 06:02 | 13:02 | 2.1326 | 11:02 | 27:04 | 12:02 | 1.6699 |
| 02:01 | 15:02 | 40:01 | 2.1320 | 02:10 | 40:06 | 12:01 | 1.6389 |
| 02:03 | 01:02 | 46:01 | 2.1255 | 02:01 | 15:11 | 15:01 | 1.6387 |
| 02:01 | 08:01 | 15:18 | 2.1153 | 11:01 | 40:06 | 09:01 | 1.5081 |
| 24:02 | 03:03 | 40:02 | 2.1084 | 11:01 | 55:02 | 04:05 | 1.5048 |
| 02:01 | 08:03 | 48:01 | 2.0551 | 24:02 | 13:02 | 07:01 | 1.4765 |
| 02:01 | 07:02 | 38:02 | 2.0222 | 29:01 | 07:05 | 08:03 | 1.4678 |
| 02:01 | 08:22 | 40:06 | 1.9951 | 02:01 | 54:01 | 04:05 | 1.4583 |
| 02:01 | 15:02 | 51:01 | 1.9720 | 02:07 | 46:01 | 16:02 | 1.4535 |
| 11:01 | 01:02 | 15:01 | 1.9636 | 02:06 | 51:01 | 09:01 | 1.4405 |
| 02:06 | 01:02 | 46:01 | 1.9187 | 24:02 | 58:01 | 03:01 | 1.4298 |
| 24:02 | 01:02 | 15:01 | 1.8906 | 24:02 | 15:01 | 09:01 | 1.4240 |
| 02:01 | 12:02 | 52:01 | 1.8856 | 11:01 | 13:02 | 07:01 | 1.4220 |
| 24:02 | 01:02 | 55:02 | 1.8691 | 11:01 | 15:01 | 09:01 | 1.4206 |
| 02:01 | 01:02 | 15:01 | 1.8622 | 11:01 | 35:01 | 15:01 | 1.4181 |
| 24:02 | 04:03 | 15:25 | 1.8182 | 24:02 | 15:25 | 12:02 | 1.4160 |
| 02:03 | 03:04 | 13:01 | 1.8081 | 02:07 | 46:01 | 12:01 | 1.3979 |
| 02:01 | 08:01 | 48:01 | 1.7904 | 24:02 | 46:01 | 08:03 | 1.3967 |
| 11:01 | 07:02 | 52:01 | 1.7790 | 24:02 | 51:01 | 15:01 | 1.3777 |
| 03:01 | 12:03 | 35:03 | 1.7727 | 26:01 | 08:01 | 03:01 | 1.3397 |
| 01:01 | 12:02 | 52:01 | 1.7675 | 01:01 | 52:01 | 15:02 | 1.3394 |
| 11:01 | 15:02 | 51:02 | 1.7627 | 02:03 | 52:01 | 14:04 | 1.3388 |
| 24:02 | 15:02 | 51:02 | 1.7341 | 03:01 | 52:01 | 15:02 | 1.3340 |
| 02:01 | 08:01 | 15:01 | 1.7203 | 11:01 | 40:01 | 14:54 | 1.3185 |
| 02:03 | 07:02 | 40:01 | 1.7164 | 11:01 | 55:02 | 12:02 | 1.3178 |
| 02:06 | 01:02 | 54:01 | 1.7103 | 33:03 | 58:01 | 09:01 | 1.3132 |
| 02:01 | 04:01 | 15:01 | 1.7089 | 02:01 | 40:01 | 08:03 | 1.2745 |
| 02:01 | 03:02 | 58:01 | 1.6954 | 01:01 | 08:01 | 03:01 | 1.2709 |
| 11:01 | 04:01 | 35:01 | 1.6914 | 24:02 | 54:01 | 09:01 | 1.2685 |
| 24:02 | 06:02 | 13:02 | 1.6846 | 30:01 | 13:02 | 09:01 | 1.2576 |
| 24:02 | 12:02 | 52:01 | 1.6558 | 02:01 | 48:01 | 15:01 | 1.2543 |
| 11:01 | 08:01 | 48:01 | 1.6363 | 02:01 | 52:01 | 15:02 | 1.2495 |
| 11:02 | 07:02 | 40:01 | 1.6115 | 33:01 | 14:02 | 01:02 | 1.2424 |
| 31:01 | 15:02 | 51:01 | 1.6104 | 02:01 | 15:01 | 11:01 | 1.2416 |
| 24:02 | 15:02 | 51:01 | 1.6072 | 11:01 | 40:01 | 04:03 | 1.2270 |
| 11:01 | 06:02 | 13:02 | 1.5980 | 11:01 | 15:32 | 15:04 | 1.2243 |
| 24:02 | 03:03 | 15:11 | 1.5963 | 02:01 | 67:01 | 16:02 | 1.2218 |
| 26:01 | 07:02 | 08:01 | 1.5875 | 33:03 | 58:01 | 15:01 | 1.2180 |
| 02:07 | 03:04 | 13:01 | 1.5647 | 02:01 | 35:01 | 15:01 | 1.2089 |
| 02:07 | 14:02 | 51:01 | 1.5477 | 11:01 | 51:01 | 15:01 | 1.1904 |
| 11:01 | 03:03 | 15:11 | 1.5417 | 11:01 | 13:01 | 09:01 | 1.1858 |
| 11:01 | 07:02 | 13:01 | 1.5415 | 24:02 | 40:02 | 15:01 | 1.1575 |
| 11:01 | 04:03 | 15:25 | 1.5400 | 30:01 | 13:02 | 15:01 | 1.1439 |
| 33:03 | 01:02 | 46:01 | 1.5267 | 24:02 | 54:01 | 14:05 | 1.1420 |
| 11:02 | 01:02 | 46:01 | 1.5266 | 11:01 | 51:01 | 11:01 | 1.1358 |
| 02:06 | 03:04 | 40:01 | 1.5079 | 02:01 | 46:01 | 08:03 | 1.1349 |
| 02:01 | 01:02 | 55:02 | 1.5004 | 24:02 | 40:01 | 16:02 | 1.1247 |
| 11:01 | 12:03 | 55:02 | 1.4664 | 26:01 | 38:01 | 01:01 | 1.1236 |
| 11:01 | 12:03 | 15:32 | 1.4648 | 30:01 | 13:02 | 11:01 | 1.1109 |
| 02:07 | 08:01 | 15:02 | 1.4629 | 03:01 | 35:03 | 07:01 | 1.1081 |
| 24:02 | 03:03 | 15:07 | 1.4621 | 02:06 | 46:01 | 09:01 | 1.0794 |
| 33:01 | 08:02 | 14:02 | 1.4512 | 24:02 | 35:01 | 09:01 | 1.0703 |
| 02:06 | 08:01 | 15:02 | 1.4486 | 11:01 | 15:01 | 11:01 | 1.0562 |
| 02:03 | 07:02 | 52:01 | 1.4337 | 11:01 | 46:01 | 14:54 | 1.0481 |
| 24:07 | 04:01 | 35:05 | 1.4286 | 11:01 | 38:02 | 12:02 | 1.0479 |
| 31:01 | 04:01 | 35:01 | 1.4018 | 02:07 | 40:01 | 09:01 | 1.0422 |
| 02:06 | 08:22 | 48:01 | 1.3813 | 02:06 | 51:01 | 12:01 | 1.0412 |
| 24:02 | 04:01 | 40:01 | 1.3780 | 11:01 | 55:02 | 09:01 | 1.0289 |
| 02:03 | 12:03 | 55:02 | 1.3765 | 24:02 | 40:01 | 12:01 | 1.0285 |
| 11:01 | 15:02 | 51:01 | 1.3685 | 02:03 | 46:01 | 09:01 | 1.0255 |
| 02:03 | 08:01 | 15:02 | 1.3570 | 33:03 | 58:01 | 07:01 | 1.0213 |
| 26:01 | 07:02 | 40:01 | 1.3546 | 11:01 | 52:01 | 12:02 | 1.0163 |
| 11:01 | 08:01 | 15:18 | 1.3386 | 11:01 | 38:02 | 08:03 | 1.0138 |
| 03:01 | 04:01 | 35:01 | 1.3360 |  |  |  |  |
| 02:06 | 03:04 | 13:01 | 1.2794 |  |  |  |  |
| 02:06 | 07:02 | 40:01 | 1.2544 |  |  |  |  |
| 11:01 | 03:04 | 40:02 | 1.2539 |  |  |  |  |
| 01:01 | 07:02 | 08:01 | 1.2438 |  |  |  |  |
| 02:01 | 07:02 | 15:01 | 1.2278 |  |  |  |  |
| 02:01 | 08:01 | 15:02 | 1.2251 |  |  |  |  |
| 31:01 | 03:04 | 40:02 | 1.2123 |  |  |  |  |
| 02:07 | 03:04 | 40:01 | 1.2104 |  |  |  |  |
| 01:01 | 07:01 | 15:17 | 1.1960 |  |  |  |  |
| 02:06 | 03:03 | 15:01 | 1.1675 |  |  |  |  |
| 02:01 | 05:01 | 44:02 | 1.1627 |  |  |  |  |
| 26:01 | 01:02 | 46:01 | 1.1609 |  |  |  |  |
| 24:02 | 07:02 | 67:01 | 1.1409 |  |  |  |  |
| 24:02 | 08:01 | 15:18 | 1.1311 |  |  |  |  |
| 02:03 | 03:04 | 40:01 | 1.1310 |  |  |  |  |
| 11:01 | 12:02 | 27:04 | 1.1305 |  |  |  |  |
| 02:06 | 07:02 | 39:01 | 1.1283 |  |  |  |  |
| 24:02 | 04:01 | 35:01 | 1.1178 |  |  |  |  |
| 11:01 | 04:01 | 15:05 | 1.1167 |  |  |  |  |
| 01:01 | 07:02 | 07:02 | 1.1069 |  |  |  |  |
| 31:01 | 01:02 | 46:01 | 1.0993 |  |  |  |  |
| 11:02 | 08:01 | 15:02 | 1.0854 |  |  |  |  |
| 24:02 | 07:02 | 08:01 | 1.0738 |  |  |  |  |
| 23:01 | 04:01 | 44:03 | 1.0730 |  |  |  |  |
| 11:01 | 01:02 | 56:01 | 1.0683 |  |  |  |  |
| 02:01 | 03:04 | 40:02 | 1.0644 |  |  |  |  |
| 26:01 | 03:03 | 35:01 | 1.0480 |  |  |  |  |
| 26:01 | 03:04 | 40:06 | 1.0325 |  |  |  |  |
| 02:06 | 08:01 | 48:01 | 1.0241 |  |  |  |  |
| 32:01 | 04:01 | 35:03 | 1.0224 |  |  |  |  |
| 02:07 | 03:02 | 58:01 | 1.0208 |  |  |  |  |
| 02:01 | 07:02 | 08:01 | 1.0142 |  |  |  |  |
| 01:01 | 06:02 | 13:02 | 1.0009 |  |  |  |  |
