# Supplementary material for: High-Resolution Analyses of Human Leukocyte Antigens Allele and Haplotype Frequencies Based on 169,995 Volunteers from the China Bone Marrow Donor Registry Program
Source: PLoS One. 2015 Sep 30;10(9):e0139485. doi: 10.1371/journal.pone.0139485 (PMC4589403; doi:10.1371/journal.pone.0139485)
Supplement: S8 Table — (DOCX) [file pone.0139485.s008.docx]

**Supporting information**

**S8 Table.** Common (freq.>1‰) HLA four-locus haplotypes among the 169,995 CMDP registry donors

| HLA-A-C-B-DRB1 | | | | | HLA-A-C-B-DRB1 | | | | |
| --- | --- | --- | --- | --- | --- | --- | --- | --- | --- |
| HLA-A | HLA-C | HLA-B | HLA-DRB1 | Freq (‰) | HLA-A | HLA-C | HLA-B | HLA-DRB1 | Freq (‰) |
| 30:01 | 06:02 | 13:02 | 07:01 | 37.0778 | 24:02 | 08:01 | 15:02 | 12:02 | 1.7236 |
| 02:07 | 01:02 | 46:01 | 09:01 | 24.7374 | 11:01 | 07:02 | 40:01 | 04:05 | 1.7185 |
| 33:03 | 03:02 | 58:01 | 03:01 | 23.9421 | 02:07 | 01:02 | 46:01 | 11:01 | 1.6996 |
| 11:01 | 08:01 | 15:02 | 12:02 | 11.6531 | 24:02 | 03:04 | 40:01 | 09:01 | 1.6991 |
| 33:03 | 03:02 | 58:01 | 13:02 | 10.6997 | 11:02 | 12:02 | 27:04 | 12:02 | 1.6649 |
| 02:07 | 01:02 | 46:01 | 08:03 | 9.3773 | 24:02 | 07:02 | 07:02 | 15:01 | 1.6599 |
| 33:03 | 14:03 | 44:03 | 13:02 | 7.4719 | 29:01 | 15:05 | 07:05 | 10:01 | 1.6502 |
| 11:01 | 03:04 | 13:01 | 15:01 | 7.0266 | 02:01 | 14:02 | 51:01 | 09:01 | 1.6412 |
| 01:01 | 06:02 | 37:01 | 10:01 | 6.6434 | 02:06 | 08:01 | 40:06 | 09:01 | 1.6365 |
| 11:01 | 01:02 | 46:01 | 09:01 | 5.8669 | 11:01 | 03:04 | 40:01 | 11:01 | 1.6114 |
| 02:01 | 03:04 | 13:01 | 12:02 | 5.7895 | 02:10 | 08:01 | 40:06 | 12:01 | 1.6113 |
| 11:01 | 04:01 | 15:01 | 04:06 | 5.5946 | 02:01 | 03:03 | 15:11 | 15:01 | 1.5975 |
| 11:01 | 08:01 | 15:02 | 15:01 | 4.9617 | 24:02 | 07:02 | 40:01 | 08:03 | 1.5743 |
| 01:01 | 06:02 | 57:01 | 07:01 | 4.8409 | 24:02 | 04:01 | 15:01 | 04:06 | 1.5524 |
| 33:03 | 07:06 | 44:03 | 07:01 | 4.6713 | 29:01 | 15:05 | 07:05 | 08:03 | 1.4615 |
| 24:02 | 01:02 | 54:01 | 04:05 | 4.4675 | 24:02 | 03:03 | 35:01 | 15:01 | 1.4425 |
| 11:01 | 07:02 | 40:01 | 08:03 | 4.2446 | 24:02 | 04:03 | 15:25 | 12:02 | 1.3605 |
| 02:07 | 01:02 | 46:01 | 14:54 | 4.2142 | 02:01 | 01:02 | 54:01 | 04:05 | 1.3596 |
| 11:01 | 03:04 | 13:01 | 12:02 | 3.7975 | 24:02 | 06:02 | 13:02 | 07:01 | 1.3497 |
| 11:01 | 07:02 | 40:01 | 09:01 | 3.7383 | 03:01 | 12:02 | 52:01 | 15:02 | 1.3250 |
| 02:03 | 07:02 | 38:02 | 16:02 | 3.6699 | 01:01 | 12:02 | 52:01 | 15:02 | 1.3237 |
| 02:01 | 03:03 | 15:11 | 09:01 | 3.6479 | 02:07 | 01:02 | 46:01 | 16:02 | 1.3192 |
| 11:01 | 14:02 | 51:01 | 09:01 | 3.1926 | 24:02 | 07:02 | 40:01 | 09:01 | 1.3136 |
| 32:01 | 12:02 | 52:01 | 15:02 | 3.1893 | 11:01 | 03:04 | 40:01 | 09:01 | 1.3084 |
| 24:02 | 01:02 | 46:01 | 09:01 | 3.1862 | 26:01 | 07:02 | 08:01 | 03:01 | 1.3054 |
| 24:02 | 14:02 | 51:01 | 09:01 | 3.1568 | 02:06 | 14:02 | 51:01 | 09:01 | 1.3028 |
| 11:01 | 07:02 | 07:02 | 01:01 | 2.8979 | 11:01 | 06:02 | 13:02 | 07:01 | 1.3019 |
| 03:01 | 07:02 | 07:02 | 15:01 | 2.8170 | 24:02 | 03:02 | 58:01 | 03:01 | 1.3018 |
| 24:02 | 08:01 | 40:06 | 09:01 | 2.6406 | 24:02 | 01:02 | 46:01 | 08:03 | 1.3004 |
| 11:01 | 12:02 | 52:01 | 15:02 | 2.5516 | 02:01 | 07:02 | 40:01 | 09:01 | 1.2624 |
| 11:01 | 03:04 | 13:01 | 16:02 | 2.4837 | 02:03 | 07:02 | 52:01 | 14:04 | 1.2613 |
| 03:01 | 05:01 | 44:02 | 13:01 | 2.4392 | 33:03 | 03:02 | 58:01 | 09:01 | 1.2548 |
| 02:01 | 01:02 | 46:01 | 09:01 | 2.4321 | 02:01 | 08:01 | 40:06 | 09:01 | 1.2451 |
| 24:02 | 03:04 | 13:01 | 12:02 | 2.4060 | 11:01 | 01:02 | 55:02 | 04:05 | 1.2428 |
| 02:07 | 01:02 | 46:01 | 15:01 | 2.3792 | 33:01 | 08:02 | 14:02 | 01:02 | 1.2362 |
| 11:01 | 01:02 | 54:01 | 04:05 | 2.3649 | 02:07 | 01:02 | 46:01 | 12:01 | 1.2244 |
| 24:02 | 03:04 | 40:01 | 11:01 | 2.3393 | 24:02 | 01:02 | 54:01 | 09:01 | 1.2239 |
| 02:07 | 01:02 | 46:01 | 12:02 | 2.3221 | 02:01 | 12:02 | 52:01 | 15:02 | 1.2022 |
| 32:01 | 04:01 | 44:03 | 07:01 | 2.2559 | 11:01 | 08:01 | 40:06 | 09:01 | 1.1988 |
| 11:01 | 07:02 | 40:01 | 15:01 | 2.2219 | 02:01 | 07:02 | 67:01 | 16:02 | 1.1962 |
| 02:03 | 07:02 | 38:02 | 08:03 | 2.1074 | 30:01 | 06:02 | 13:02 | 09:01 | 1.1946 |
| 02:05 | 06:02 | 50:01 | 07:01 | 2.0823 | 33:03 | 03:02 | 58:01 | 15:01 | 1.1749 |
| 24:02 | 03:04 | 13:01 | 15:01 | 2.0157 | 11:01 | 12:03 | 15:32 | 15:04 | 1.1606 |
| 24:02 | 03:04 | 40:01 | 15:01 | 1.9847 | 24:02 | 14:02 | 51:01 | 15:01 | 1.1372 |
| 11:01 | 07:02 | 40:01 | 11:01 | 1.9824 | 26:01 | 12:03 | 38:01 | 01:01 | 1.1171 |
| 31:01 | 03:03 | 15:01 | 15:01 | 1.9493 | 30:01 | 06:02 | 13:02 | 11:01 | 1.1067 |
| 11:01 | 03:02 | 58:01 | 03:01 | 1.8860 | 03:01 | 12:03 | 35:03 | 07:01 | 1.1036 |
| 02:03 | 07:02 | 38:02 | 15:02 | 1.8700 | 01:01 | 07:02 | 08:01 | 03:01 | 1.0921 |
| 11:01 | 07:02 | 40:01 | 12:02 | 1.8498 | 30:01 | 06:02 | 13:02 | 15:01 | 1.0861 |
| 02:07 | 01:03 | 46:01 | 09:01 | 1.8483 | 24:02 | 01:02 | 54:01 | 14:05 | 1.0837 |
| 11:01 | 01:02 | 46:01 | 08:03 | 1.8281 | 24:02 | 08:03 | 48:01 | 15:01 | 1.0725 |
| 11:01 | 07:02 | 40:01 | 12:01 | 1.8210 | 11:01 | 03:03 | 35:01 | 15:01 | 1.0511 |
| 11:01 | 07:02 | 39:01 | 08:03 | 1.8000 | 11:01 | 07:02 | 38:02 | 12:02 | 1.0316 |
| 24:02 | 04:01 | 15:27 | 04:06 | 1.7813 | 02:01 | 01:02 | 46:01 | 08:03 | 1.0307 |
| 02:07 | 01:02 | 46:01 | 04:05 | 1.7636 | 02:01 | 03:03 | 15:01 | 15:01 | 1.0224 |
| 02:01 | 06:02 | 13:02 | 07:01 | 1.7364 | 33:03 | 03:02 | 58:01 | 07:01 | 1.0140 |
| 11:01 | 03:04 | 40:01 | 15:01 | 1.7311 | 02:01 | 03:03 | 35:01 | 15:01 | 1.0093 |
