# Supplementary material for: High-Resolution Analyses of Human Leukocyte Antigens Allele and Haplotype Frequencies Based on 169,995 Volunteers from the China Bone Marrow Donor Registry Program
Source: PLoS One. 2015 Sep 30;10(9):e0139485. doi: 10.1371/journal.pone.0139485 (PMC4589403; doi:10.1371/journal.pone.0139485)
Supplement: S9 Table — (DOCX) [file pone.0139485.s009.docx]

**Supporting information**

**S9 Table.** Common (freq.>1‰) HLA five-loci haplotypes among the 169,995 CMDP registry donors

| HLA-A-C-B-DRB1-DQB1 | | | | | | HLA-A-C-B-DRB1-DQB1 | | | | | |
| --- | --- | --- | --- | --- | --- | --- | --- | --- | --- | --- | --- |
| A | C | B | DRB1 | DQB1 | Freq.(‰) | A | C | B | DRB1 | DQB1 | Freq.(‰) |
| 30:01 | 06:02 | 13:02 | 07:01 | 02:02 | 37.0023 | 02:01 | 14:02 | 51:01 | 09:01 | 03:03 | 1.6304 |
| 02:07 | 01:02 | 46:01 | 09:01 | 03:03 | 24.6414 | 02:06 | 08:01 | 40:06 | 09:01 | 03:03 | 1.6140 |
| 33:03 | 03:02 | 58:01 | 03:01 | 02:01 | 24.0021 | 24:02 | 03:04 | 40:01 | 09:01 | 03:03 | 1.6113 |
| 11:01 | 08:01 | 15:02 | 12:02 | 03:01 | 11.2726 | 02:07 | 01:02 | 46:01 | 04:05 | 04:01 | 1.5989 |
| 33:03 | 03:02 | 58:01 | 13:02 | 06:09 | 10.6212 | 11:01 | 03:04 | 40:01 | 11:01 | 03:01 | 1.5806 |
| 02:07 | 01:02 | 46:01 | 08:03 | 06:01 | 9.2546 | 02:10 | 08:01 | 40:06 | 12:01 | 03:01 | 1.5649 |
| 33:03 | 14:03 | 44:03 | 13:02 | 06:04 | 7.3898 | 11:01 | 07:02 | 40:01 | 04:05 | 04:01 | 1.5415 |
| 01:01 | 06:02 | 37:01 | 10:01 | 05:01 | 6.6029 | 24:02 | 04:01 | 15:01 | 04:06 | 03:02 | 1.5402 |
| 11:01 | 03:04 | 13:01 | 15:01 | 06:01 | 6.3828 | 24:02 | 07:02 | 40:01 | 08:03 | 06:01 | 1.5254 |
| 02:01 | 03:04 | 13:01 | 12:02 | 03:01 | 5.7905 | 31:01 | 03:03 | 15:01 | 15:01 | 06:02 | 1.5185 |
| 11:01 | 01:02 | 46:01 | 09:01 | 03:03 | 5.7645 | 02:07 | 01:02 | 46:01 | 11:01 | 03:01 | 1.5048 |
| 11:01 | 04:01 | 15:01 | 04:06 | 03:02 | 5.5765 | 02:03 | 07:02 | 38:02 | 15:02 | 05:01 | 1.5042 |
| 33:03 | 07:06 | 44:03 | 07:01 | 02:02 | 4.6531 | 29:01 | 15:05 | 07:05 | 08:03 | 03:01 | 1.4084 |
| 01:01 | 06:02 | 57:01 | 07:01 | 03:03 | 4.5028 | 24:02 | 03:03 | 35:01 | 15:01 | 06:02 | 1.3956 |
| 11:01 | 08:01 | 15:02 | 15:01 | 06:01 | 4.3962 | 24:02 | 04:03 | 15:25 | 12:02 | 03:01 | 1.3520 |
| 24:02 | 01:02 | 54:01 | 04:05 | 04:01 | 4.3849 | 11:01 | 07:02 | 40:01 | 15:01 | 06:02 | 1.3463 |
| 11:01 | 07:02 | 40:01 | 08:03 | 06:01 | 4.2056 | 02:01 | 01:02 | 54:01 | 04:05 | 04:01 | 1.3255 |
| 02:07 | 01:02 | 46:01 | 14:54 | 05:02 | 3.8003 | 01:01 | 12:02 | 52:01 | 15:02 | 06:01 | 1.3202 |
| 11:01 | 03:04 | 13:01 | 12:02 | 03:01 | 3.6721 | 02:07 | 01:02 | 46:01 | 16:02 | 05:02 | 1.3136 |
| 02:03 | 07:02 | 38:02 | 16:02 | 05:02 | 3.6439 | 03:01 | 12:02 | 52:01 | 15:02 | 06:01 | 1.3077 |
| 11:01 | 07:02 | 40:01 | 09:01 | 03:03 | 3.5921 | 24:02 | 06:02 | 13:02 | 07:01 | 02:02 | 1.3041 |
| 02:01 | 03:03 | 15:11 | 09:01 | 03:03 | 3.5506 | 26:01 | 07:02 | 08:01 | 03:01 | 02:01 | 1.2977 |
| 32:01 | 12:02 | 52:01 | 15:02 | 06:01 | 3.1758 | 24:02 | 03:02 | 58:01 | 03:01 | 02:01 | 1.2853 |
| 24:02 | 01:02 | 46:01 | 09:01 | 03:03 | 3.1361 | 24:02 | 01:02 | 46:01 | 08:03 | 06:01 | 1.2835 |
| 11:01 | 14:02 | 51:01 | 09:01 | 03:03 | 3.1353 | 24:02 | 07:02 | 40:01 | 09:01 | 03:03 | 1.2760 |
| 24:02 | 14:02 | 51:01 | 09:01 | 03:03 | 3.1312 | 02:03 | 07:02 | 52:01 | 14:04 | 05:03 | 1.2613 |
| 11:01 | 07:02 | 07:02 | 01:01 | 05:01 | 2.8888 | 02:01 | 07:02 | 40:01 | 09:01 | 03:03 | 1.2526 |
| 03:01 | 07:02 | 07:02 | 15:01 | 06:02 | 2.8423 | 24:02 | 03:04 | 13:01 | 15:01 | 06:01 | 1.2484 |
| 24:02 | 08:01 | 40:06 | 09:01 | 03:03 | 2.6254 | 11:01 | 03:04 | 40:01 | 09:01 | 03:03 | 1.2452 |
| 11:01 | 12:02 | 52:01 | 15:02 | 06:01 | 2.5684 | 11:01 | 06:02 | 13:02 | 07:01 | 02:02 | 1.2397 |
| 11:01 | 03:04 | 13:01 | 16:02 | 05:02 | 2.4453 | 33:01 | 08:02 | 14:02 | 01:02 | 05:01 | 1.2238 |
| 03:01 | 05:01 | 44:02 | 13:01 | 06:03 | 2.4337 | 02:01 | 08:01 | 40:06 | 09:01 | 03:03 | 1.2206 |
| 24:02 | 03:04 | 13:01 | 12:02 | 03:01 | 2.3835 | 02:06 | 14:02 | 51:01 | 09:01 | 03:03 | 1.2194 |
| 11:01 | 01:02 | 54:01 | 04:05 | 04:01 | 2.3426 | 24:02 | 01:02 | 54:01 | 09:01 | 03:03 | 1.2148 |
| 02:01 | 01:02 | 46:01 | 09:01 | 03:03 | 2.2951 | 24:02 | 03:04 | 40:01 | 15:01 | 06:02 | 1.2095 |
| 24:02 | 03:04 | 40:01 | 11:01 | 03:01 | 2.2907 | 33:03 | 03:02 | 58:01 | 09:01 | 03:03 | 1.1959 |
| 32:01 | 04:01 | 44:03 | 07:01 | 02:02 | 2.2347 | 11:01 | 01:02 | 55:02 | 04:05 | 04:01 | 1.1833 |
| 02:07 | 01:02 | 46:01 | 12:02 | 03:01 | 2.1140 | 02:01 | 12:02 | 52:01 | 15:02 | 06:01 | 1.1797 |
| 02:03 | 07:02 | 38:02 | 08:03 | 06:01 | 2.0931 | 02:01 | 07:02 | 67:01 | 16:02 | 05:02 | 1.1763 |
| 02:05 | 06:02 | 50:01 | 07:01 | 02:02 | 2.0617 | 02:07 | 01:02 | 46:01 | 15:01 | 06:02 | 1.1730 |
| 11:01 | 07:02 | 40:01 | 11:01 | 03:01 | 1.9728 | 02:01 | 03:03 | 15:11 | 15:01 | 06:02 | 1.1579 |
| 11:01 | 03:02 | 58:01 | 03:01 | 02:01 | 1.8310 | 11:01 | 12:03 | 15:32 | 15:04 | 05:02 | 1.1522 |
| 02:07 | 01:03 | 46:01 | 09:01 | 03:03 | 1.8267 | 11:01 | 08:01 | 40:06 | 09:01 | 03:03 | 1.1500 |
| 11:01 | 01:02 | 46:01 | 08:03 | 06:01 | 1.8155 | 30:01 | 06:02 | 13:02 | 09:01 | 03:03 | 1.1191 |
| 11:01 | 07:02 | 39:01 | 08:03 | 06:01 | 1.7718 | 26:01 | 12:03 | 38:01 | 01:01 | 05:01 | 1.1088 |
| 24:02 | 04:01 | 15:27 | 04:06 | 03:02 | 1.7613 | 03:01 | 12:03 | 35:03 | 07:01 | 03:03 | 1.0922 |
| 11:01 | 07:02 | 40:01 | 12:02 | 03:01 | 1.7551 | 01:01 | 07:02 | 08:01 | 03:01 | 02:01 | 1.0871 |
| 11:01 | 07:02 | 40:01 | 12:01 | 03:01 | 1.7224 | 02:07 | 01:02 | 46:01 | 12:01 | 03:01 | 1.0870 |
| 02:01 | 06:02 | 13:02 | 07:01 | 02:02 | 1.6987 | 24:02 | 01:02 | 54:01 | 14:05 | 05:03 | 1.0727 |
| 24:02 | 08:01 | 15:02 | 12:02 | 03:01 | 1.6794 | 30:01 | 06:02 | 13:02 | 11:01 | 03:01 | 1.0696 |
| 24:02 | 07:02 | 07:02 | 15:01 | 06:02 | 1.6741 | 02:01 | 01:02 | 46:01 | 08:03 | 06:01 | 1.0177 |
| 29:01 | 15:05 | 07:05 | 10:01 | 05:01 | 1.6470 | 11:01 | 03:03 | 35:01 | 15:01 | 06:02 | 1.0038 |
| 11:02 | 12:02 | 27:04 | 12:02 | 03:01 | 1.6313 |  |  |  |  |  |  |
