# Supplementary material for: High-Resolution Analyses of Human Leukocyte Antigens Allele and Haplotype Frequencies Based on 169,995 Volunteers from the China Bone Marrow Donor Registry Program
Source: PLoS One. 2015 Sep 30;10(9):e0139485. doi: 10.1371/journal.pone.0139485 (PMC4589403; doi:10.1371/journal.pone.0139485)
Supplement: S10 Table — (DOCX) [file pone.0139485.s010.docx]

**Supporting information**

**S10 Table.** The numbers of donors from each of the geographic regions of China

| Number | NE | NC | NW | EC | CC | SC | SW |
| --- | --- | --- | --- | --- | --- | --- | --- |
| Number of donors observed  in this study | 12493 | 27819 | 14409 | 51132 | 24432 | 18657 | 21053 |
| Number of resident population  in 2010 ^a^ (million) | 109.5 | 164.8 | 96.6 | 392.9 | 216.9 | 159.0 | 192.9 |
| Ratio (donors/resident population) | 1.1/10^4^ | 1.7/10^4^ | 1.5/10^4^ | 1.3/10^4^ | 1.1/10^4^ | 1.2/10^4^ | 1.1/10^4^ |

a: from the 6^th^ National Population Census

(National Bureau of Statistics of China, <http://www.stats.gov.cn/tjsj/pcsj/rkpc/6rp/indexch.htm> )
