# Supplementary material for: High-Resolution Analyses of Human Leukocyte Antigens Allele and Haplotype Frequencies Based on 169,995 Volunteers from the China Bone Marrow Donor Registry Program
Source: PLoS One. 2015 Sep 30;10(9):e0139485. doi: 10.1371/journal.pone.0139485 (PMC4589403; doi:10.1371/journal.pone.0139485)
Supplement: S11 Table — (DOCX) [file pone.0139485.s011.docx]

**Supporting information**

**S11 Table. Frequencies** of several distinct haplotypes in each of the geographic regions

| NO. | A-C-B-DRB1-DQB1 haplotype | Frequency (‰) in each region | | | | | | |
| --- | --- | --- | --- | --- | --- | --- | --- | --- |
|  |  | **NE** | **NC** | **NW** | **EC** | **CC** | **SC** | **SW** |
| 20^th^ | A*02:03-C*07:02-B*38:02-DRB1*16:02-DQB1*05:02 | 1.15 | 1.73 | 1.35 | 3.22 | 3.78 | **10.11** | 3.45 |
| 31^st^ | A*11:01-C*03:04-B*13:01-DRB1*16:02-DQB1*05:02 | 0.59 | 0.83 | 1.23 | 1.91 | 2.17 | **7.71** | 2.88 |
| 81^st^ | A*24:02-C*03:04-B*13:01-DRB1*15:01-DQB1*06:01 | 0.45 | 0.48 | 0.37 | 0.91 | 1.42 | **3.91** | 1.62 |
| 68^th^ | A*24:02-C*04:03-B*15:25-DRB1*12:02-DQB1*03:01 | 0.39 | 0.34 | 0.47 | 0.36 | 0.20 | 0.72 | **7.90** |
| 79^th^ | A*02:03-C*07:02-B*52:01-DRB1*14:04-DQB1*05:03 | 1.26 | 0.71 | 0.30 | 0.85 | 0.45 | 0.29 | **5.11** |
| 95^th^ | A*11:01-C*12:03-B*15:32-DRB1*15:04-DQB1*05:02 | 0.71 | 1.16 | 1.59 | 0.32 | 0.64 | 0.27 | **4.05** |
| 18^th^ | A*02:07-C*01:02-B*46:01-DRB1*14:54-DQB1*05:02 | 0.78 | 1.26 | 3.00 | 1.97 | 3.51 | **7.49** | **10.14** |
| 94^th^ | A*02:01-C*03:03-B*15:11-DRB1*15:01-DQB1*06:02 | **2.01** | **2.34** | 0.93 | 0.98 | 0.81 | 0.29 | 0.62 |
| 40^th^ | A*02:05-C*06:02-B*50:01-DRB1*07:01-DQB1*02:02 | **3.15** | **3.40** | **3.42** | 1.51 | 1.55 | 0.66 | 1.29 |
