# Supplementary material for: High-Resolution Analyses of Human Leukocyte Antigens Allele and Haplotype Frequencies Based on 169,995 Volunteers from the China Bone Marrow Donor Registry Program
Source: PLoS One. 2015 Sep 30;10(9):e0139485. doi: 10.1371/journal.pone.0139485 (PMC4589403; doi:10.1371/journal.pone.0139485)
Supplement: S12 Table — (DOCX) [file pone.0139485.s012.docx]

**Supporting information**

**S12 Table.** **HLA matches within each region of China**

| Group | No. of individuals | No. of Matches | Match Rate (95%CI) | Odds Ratio (95% CI) |
| --- | --- | --- | --- | --- |
| NW | 14409 | 2066 | 1.99 x 10^-5^ (1.91x10^-5^-2.08x10^-5^) | 1 |
| NC | 27819 | 8402 | 2.17 x 10^-5^ (2.13x10^-5^-2.22x10^-5^) | 1.09 (1.04-1.14)* |
| NE | 12493 | 2242 | 2.87 x10^-5^ (2.76x10^-5^-2.99x10^-5^) | 1.44 (1.36-1.53)* |
| SW | 21053 | 7091 | 3.20 x 10^-5^ (3.13x10^-5^-3.28x10^-5^) | 1.61 (1.53-1.69)* |
| CC | 24432 | 14177 | 4.75 x 10^-5^ (4.67x10^-5^-4.83x10^-5^) | 2.39 (2.28-2.50)* |
| EC | 51132 | 70878 | 5.42 x 10^-5^ (5.38x10^-5^-5.46x10^-5^) | 2.72 (2.61-2.85)* |
| SC | 18657 | 13598 | 7.81 x 10^-5^ (7.68x10^-5^-7.95x10^-5^) | 3.93 (3.75-4.11)* |

* P< 0.05 (chi-square test)
